# Modeling and insights into the structural characteristics of drug-induced autoimmune diseases

**DOI:** 10.3389/fimmu.2022.1015409

**Published:** 2022-10-24

**Authors:** Huizhu Guo, Peitao Zhang, Ruiqiu Zhang, Yuqing Hua, Pei Zhang, Xueyan Cui, Xin Huang, Xiao Li

**Affiliations:** ^1^ Department of Clinical Pharmacy, The First Affiliated Hospital of Shandong First Medical University & Shandong Provincial Qianfoshan Hospital, Shandong Engineering and Technology Research Center for Pediatric Drug Development, Shandong Medicine and Health Key Laboratory of Clinical Pharmacy, Jinan, China; ^2^ Department of Clinical Pharmacy, Shandong Provincial Qianfoshan Hospital, Shandong University, Jinan, China

**Keywords:** drug-induced autoimmune diseases, computational toxicology, machine learning, molecular fingerprinting, structural alert

## Abstract

The incidence and complexity of drug-induced autoimmune diseases (DIAD) have been on the rise in recent years, which may lead to serious or fatal consequences. Besides, many environmental and industrial chemicals can also cause DIAD. However, there are few effective approaches to estimate the DIAD potential of drugs and other chemicals currently, and the structural characteristics and mechanism of action of DIAD compounds have not been clarified. In this study, we developed the *in silico* models for chemical DIAD prediction and investigated the structural characteristics of DIAD chemicals based on the reliable drug data on human autoimmune diseases. We collected 148 medications which were reported can cause DIAD clinically and 450 medications that clearly do not cause DIAD. Several different machine learning algorithms and molecular fingerprints were combined to develop the *in silico* models. The best performed model provided the good overall accuracy on validation set with 76.26%. The model was made freely available on the website http://diad.sapredictor.cn/. To further investigate the differences in structural characteristics between DIAD chemicals and non-DIAD chemicals, several key physicochemical properties were analyzed. The results showed that AlogP, molecular polar surface area (MPSA), and the number of hydrogen bond donors (nHDon) were significantly different between the DIAD and non-DIAD structures. They may be related to the DIAD toxicity of chemicals. In addition, 14 structural alerts (SA) for DIAD toxicity were detected from predefined substructures. The SAs may be helpful to explain the mechanism of action of drug induced autoimmune disease, and can used to identify the chemicals with potential DIAD toxicity. The structural alerts have been integrated in a structural alert-based web server SApredictor (http://www.sapredictor.cn). We hope the results could provide useful information for the recognition of DIAD chemicals and the insights of structural characteristics for chemical DIAD toxicity.

## Introduction

Autoimmune diseases refer to problems where the immune system attacks healthy cells in the body by mistake (1). It was reported that there are about 20 million autoimmune disease patients in the United States, accounting for about 7% to 9% of the total population (2). Meanwhile, the incidence of autoimmune diseases in industrialized countries is also increasing in recent years with the continuous deterioration of the environment (3). Up to now, more than 100 types of autoimmune diseases have been discovered, but the treatment of autoimmune diseases can only repair the damage already caused as the main direction. Most patients need long-term or even lifelong medication, resulting in huge medical economic burden. It cost more than 100 billion US dollars every year in the USA healthcare system (4). More importantly, many patients’ conditions are dangerous, seriously affecting the quality of life, and even fatal.

The development of autoimmune diseases requires both genetic predisposition and environmental factors to jointly trigger immune pathways, which gradually develop and eventually lead to tissue destruction (5). As one of the environmental factors, medications and industrial chemicals also have been reported can interfere with human immune system and induce autoimmune diseases. For instance, drug-induced lupus accounts for about 10% of all systemic lupus cases in the USA (6). Besides, about 12-17% of autoimmune hepatitis cases were believed to be induced by clinical drugs (4). The incidence and complexity of drug-induced autoimmune diseases have been on the rise in recent years. Since sulfadiazine was first reported to cause lupus-like symptoms in 1945, more than 100 drugs have been found to cause drug-induced autoimmune diseases (DIAD). As a special type B drug reaction, DIAD is unpredictable, with an incubation period of months or even years, sometimes leading to serious or fatal consequences. Compared with primary autoimmune diseases, DIAD has more complex clinical manifestations, with significant differences in epidemiology and pathology (7).

The mechanisms of DIAD have not been fully clarified. Most DIAD chemicals are small molecules and have no immunogenicity by themselves, but these small molecule substances or their metabolites are able bind to carrier proteins and become immunogens (8). These hemicantized drugs become target antigens and induce an immune response to themselves, leading to the production of autoantibodies. However, recent studies have found that most DIAD drugs do not induce specific T cell production, but induce autoimmune response, so there may be other mechanisms for this process. The neutrophil extracellular traps (NETs) (9, 10) induced by drugs may cause or promote the occurrence of some autoimmune diseases. Therefore, the formation of NETs may also be an important cause of drug-induced autoimmune disease (11). More recently, Li, et al. reported that ferroptosis is a major factor in neutropenia and systemic autoimmune disease (12). Hence, drug-induced ferroptosis may be another possible pathway for DIAD. Many of the DIAD drugs are used for autoimmune diseases treatment themselves. These drugs may be immunostimulatory, and can act in an immunomodulatory manner under different genetic and environmental conditions (8).

Until now, there is few effective approach to estimate the DIAD potential of drugs and other chemicals (4). Computational toxicology is a structure-based, application-related management and analysis of experimental data from toxicological tests that can provide viable mechanistic explanations for the toxicity of compounds (13). This tool is particularly important in designing safe drugs and assessing environmental risks (14–24). Using computational toxicology tools to develop in silico models for chemical DIAD toxicity and analyze the structural characteristics of DIAD drugs not only helps to estimate the potential DIAD toxicity of compounds, but also helps to explore the structural basis of chemical DIAD toxicity.

In the present study, we aim to develop the machine learning models for chemical DIAD toxicity and investigate the structural characteristics of DIAD chemicals.

## Materials and methods

### Collection and preparation of DIAD and non-DIAD drugs

Only approved small molecule drugs data related to DIAD toxicity were included in this study. The DIAD drugs were extracted from two different sources: (1) the drugs with autoimmune diseases associated side effects extracted from the Side Effect Resource (SIDER) database (25); (2) positive drugs for DIAD reported in the literature (4). SIDER is a resource of adverse drug reaction (ADR), which contains the information on marketed medicines and their recorded ADRs on human. We retrieved the entire SIDER database, and extracted the ADRs related to autoimmune diseases with frequency ≥ 0.1%. The corresponding structures were obtained from the PubChem compound database (26). The negative drugs for DIAD were all extracted from Wu’s work (4). The structures were prepared by: (1) only keeping the main ingredients in mixtures; (2) excluding the inorganic and organometallic compounds; (3) converting the salts into the parent forms; (4) removing the duplicate substances. The data standardization was performed on Online Chemical Database and Modeling Environment (OCHEM) platform (27), which is a user friendly web-based platform for data exploring and modeling. The details for structure preparation can be seen in supporting information.

### Model building for chemical DIAD toxicity

As a specific artificial intelligence method, machine learning was always used for the model building which can access data and use data for automated learning (28). In this study, five commonly used machine learning methods were used for the model development, including Support Vector Machines (SVM) (29), Naive Bayes (NB) (30), K-nearest Neighbor (kNN) (31), Decision Tree (DT) (32) and Random Forest (RF) (33). These methods have been extensively used in computational toxicity studies due to the high effective and robust. The detailed descriptions for the algorithms can be learned in the corresponding literature. Herein, the SVM algorithm was performed with the LIBSVM (LIBSVM 3.16 package) (34), and the parameters for Gaussian radial basis function (RBF) kernel were optimized with a grid search method based on 5-fold cross-validation. The other algorisms were implemented in the data mining tool Orange (version 2.7, freely available at https://orange.biolab.si/orange2/). For kNN, the parameter k was also optimized based on 5-fold cross-validation. The parameters for C4.5 DT, RF and NB algorithms were optimized with the default setting in the Orange toolbox.

The molecular description was implemented with several different molecular fingerprints packages, which have been widely used in toxicity prediction of drugs and environmental chemicals. In this study, we used seven common fingerprints packages, including the Estate fingerprint (Estate, 79 bits), CDK fingerprint (FP, 1024 bits), CDK extended fingerprint (ExtendFP, 1024 bits), Klekota-Roth fingerprint (KRFP, 4860 bits), MACCS keys (MACCS, 166 bits), PubChem fingerprint (PubChem, 881 bits), and Substructure fingerprint (SubFP, 307 bits). All the fingerprints were calculated with PaDEL Descriptor (35).

### Assessment of model performance

The models were both validated with 5-fold cross validation and a validation set. Several statistical parameters were calculated for the assessment of model performance, including prediction accuracy (ACC), sensitivity (SE), specificity (SP) and the Matthew’s correlation coefficient (MCC) (36), as shown with Eqs (1-4).


(1) $$ACC=\frac{TP+TN}{TP+FN+TN+FP}$$

(2) $$SE=\frac{TP}{TP+FN}$$

(3) $$SP=\frac{TN}{TN+FP}$$

(4) $$MCC=\frac{TP*TN-FP*FN}{\sqrt{\left(TP+FP\right)\left(TP+FN\right)\left(TN+FP\right)\left(TN+FN\right)}}$$

Where TP represented true positives, FP represented false positives, TN represented true negatives, and TN represented false negatives.

In addition, the receiver operating characteristic (ROC) curve was plotted, and the values of the area under the ROC curves (AUC) were also computed.

### Analysis of molecular properties for the DIAD and non-DIAD drugs

The molecular properties of compounds can play key roles in biological and toxicological activities. Eight important molecular properties were calculated with PaDEL-Descriptor package. These properties were molecular weight (MW), molecular polar surface area (MPSA), AlogP, molecular solubility (LogS), the number of hydrogen bond acceptors (nHAcc) and donors (nHDon), the number of rotatable bonds (nRotB) and the number of aromatic rings (nAR). The MW and MPSA values can reflect the size and complexity of molecules to a certain extent. AlogP and LogS are usually used to represent the chemical lipophilicity and solubility in water. The nHAcc and nHDon values represent the hydrogen bonding ability of compounds, which also can play an important role for chemical activities.

Because of disobeying the normal distribution, the data were expressed by the median and interquartile spacing and the comparison between groups adopted Wilcoxon rank sum test in this study. The p value < 0.05 was considered with statistical significance.

### Analysis of structural alerts for chemical DIAD toxicity

Structural alert (SA) was the toxophore (usually a specific substructure or fragment) which can lead to a particular toxicity endpoint. It has been widely used for toxicity research for many different toxicity endpoints (37–44). In this study, the SAs for DIAD toxicity were detected by calculating the f-score (45) and frequency ratio of each fragment from KRFP fingerprint. The f-score is a simple feature selection technique, which can measure the discrimination of two sets. The larger f-score always suggested the feature is more discriminative (45, 46). The positive rate (PR) of a substructure was defined as below:


(5) $$PR=\frac{N_{fragment\_positive}}{N_{fragment}}$$

Where N*_fragment_positive_* was the number of DIAD compounds containing the substructure, and N*_fragment_* was the total number of all the compounds containing the substructure.

## Results and discussion

### Data set analysis

**Figure 1:**
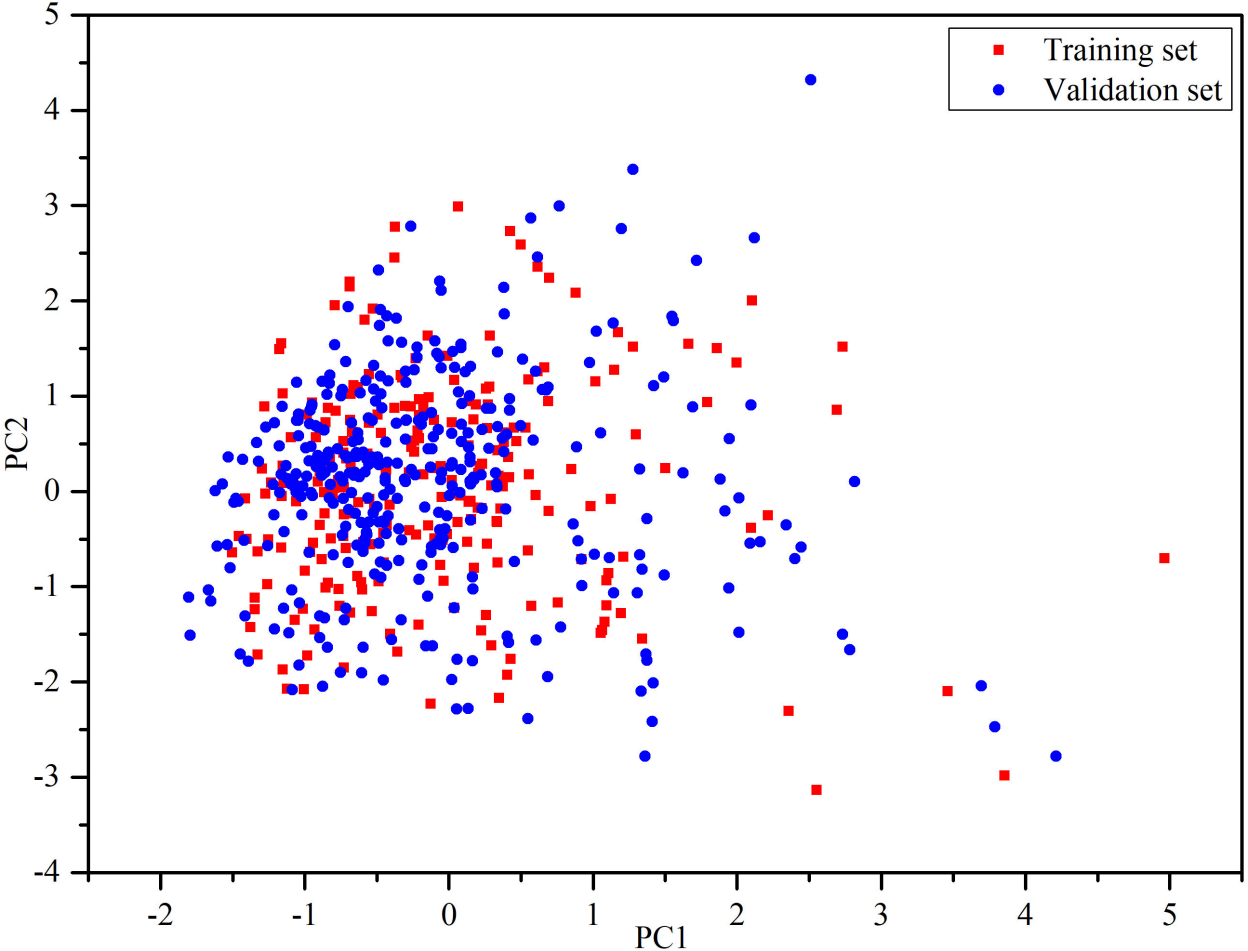
Chemical space defined by the first two principal components of physical-chemical descriptors. Red squares stand for the training set, blue circles stand for the validation set.

After filtering and preparation, there were 598 organic compounds, including 148 DIAD drugs and 450 non-DIAD drugs, extracted from the SIDER database and literature. The DIAD compounds were randomly divided into a training set and a validation set with 80%:20%. Since the non-DIAD drugs are much more than the DIAD drugs in number, and the imbalance may cause bias in model development, the non-DIAD were randomly divided into training and validation set with 25%:75%. As shown in **Table 1**, the training set contained 240 structures (115 DIAD drugs and 125 non-DIAD drugs) and the validation set contained 358 structures (33 DIAD drugs and 325 non-DIAD drugs). The structure information of the drugs can be seen in **Table S1**. The structural diversity of chemical compounds is important for the global models (47). The principal component analysis (PCA) (48) was performed based on the eight physical-chemical properties to generate the chemical space. PCA can transform data into lower dimensions from high-dimensional data, and meanwhile the trends and patterns can be retained as possible (49). Herein, the first two principal components (PC) with cumulative proportion 79.08% were kept to represent the chemical space. As shown in **Figure 1**, the results suggested that the chemical spaces of training and validation sets were similar.

**Table 1:** Detailed statistical number of drugs in the data set.

	DIAD structures	Non-DIAD structures	Total
Training set	115	125	240
Validation set	33	325	358
Total	148	450	598

### Machine learning models

There were 35 different classification models developed using the different machine learning algorithms combined with fingerprint packages. The optimized parameters for SVM and kNN models can be seen in **Table S2**. Considering the large difference in the number of DIAD and non-DIAD drugs in this study, the ACC and MCC values were paid special attention when evaluating the performance of the models, since MCC can be influenced much less by imbalanced data. Most of the models showed good performance on the 5-fold cross validation, as shown in **Table 2**. The ACC values ranged from 60.42% to 77.50%, and the MCC values ranged from 0.21 to 0.55. The model developed with SVM method and MACCS keys provided the best performance with the total accuracy 77.50%, SE value 76.52%, SP value 78.40%, AUC value 0.86 and MCC value 0.55. Besides, five models (ExtendFP_SVM, KRFP_SVM, SubFP_SVM, MACCS_kNN, and ExtendFP_kNN) also showed good predictive results on 5-fold cross validation with ACC > 75.00% and MCC > 0.50.

**Table 2:** Performances of models on 5-fold cross-validation.

Model	ACC (%)	SE (%)	SP (%)	MCC	AUC
Estate_kNN	70.83	77.39	64.80	0.42	0.77
Estate_SVM	72.08	67.83	76.00	0.44	0.79
Estate_RF	67.08	52.17	80.80	0.35	0.76
Estate_NB	65.42	66.09	64.80	0.31	0.65
Estate_CT	67.92	69.57	66.40	0.36	0.74
ExtendFP_kNN	75.42	72.17	78.40	0.51	0.82
ExtendFP_SVM	75.83	70.43	80.80	0.52	0.82
ExtendFP_RF	69.17	58.26	79.20	0.38	0.76
ExtendFP_NB	66.67	62.61	70.40	0.33	0.70
ExtendFP_CT	60.42	65.22	56.00	0.21	0.61
FP_kNN	72.92	71.3	74.40	0.46	0.81
FP_SVM	72.08	70.43	73.60	0.44	0.77
FP_RF	66.25	59.13	72.80	0.32	0.75
FP_NB	64.58	62.61	66.40	0.29	0.68
FP_CT	60.42	62.61	58.40	0.21	0.62
MACCS_kNN	77.50	80.87	74.40	0.55	0.78
MACCS_SVM	77.50	76.52	78.40	0.55	0.86
MACCS_RF	70.83	61.74	79.20	0.42	0.78
MACCS_NB	63.33	66.96	60.00	0.27	0.71
MACCS_CT	66.25	71.3	61.60	0.33	0.65
Pubchem_kNN	70.83	75.65	66.40	0.42	0.75
Pubchem_SVM	74.58	73.91	75.20	0.49	0.80
Pubchem_RF	65.42	59.13	71.20	0.31	0.75
Pubchem_NB	64.58	65.22	64.00	0.29	0.69
Pubchem_CT	67.08	66.09	68.00	0.34	0.68
SubFP_kNN	69.58	77.39	62.40	0.40	0.77
SubFP_SVM	75.42	70.43	80.00	0.51	0.78
SubFP_RF	62.08	50.43	72.80	0.24	0.71
SubFP_NB	63.75	67.83	60.00	0.28	0.69
SubFP_CT	68.75	71.3	66.40	0.38	0.69
KRFP_kNN	73.75	71.3	76.00	0.47	0.85
KRFP_SVM	77.50	73.04	81.60	0.55	0.85
KRFP_RF	62.50	25.22	96.80	0.32	0.77
KRFP_NB	67.08	65.22	68.80	0.34	0.70
KRFP_CT	70.83	73.91	68.00	0.42	0.71

**Figure 2:**
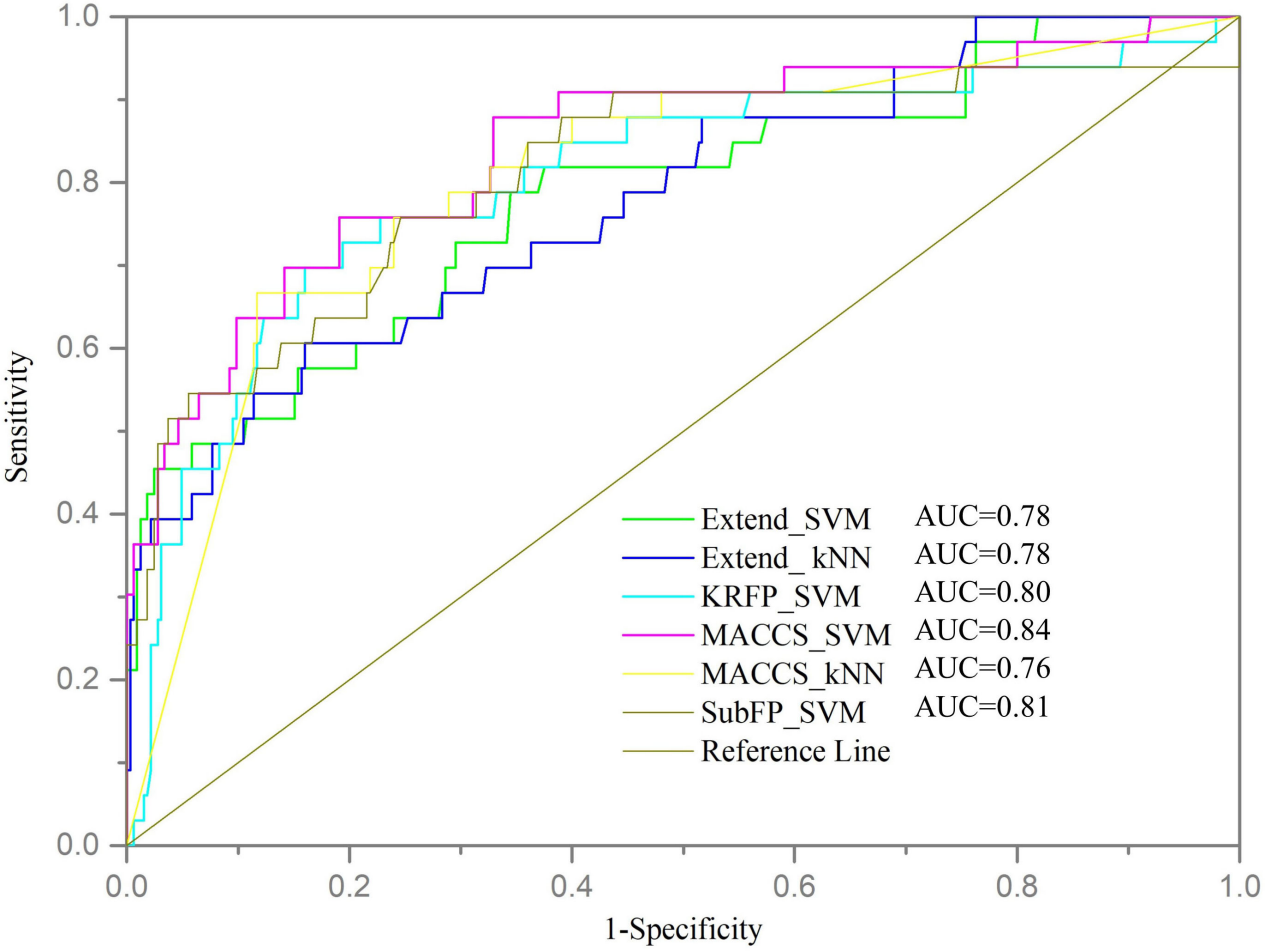
ROC curve of models on validation set. Each color line represents a model.

Furthermore, the validation set was used to assess the generalization and robustness of the six models with top performance on internal cross validation. Since the validation set was completely independent from the training set, it can be used to validate the predictive ability of models objectively. The performances of models on validation set were shown in **Table 3** and the ROC curves were shown in **Figure 2**. Most of the models, except MACCS_kNN, performed well with the ACC values higher than 75% and the MCC values over 0.25. The MACCS_SVM model also achieved good prediction accuracy of 75.98% on validation set and best AUC value of 0.84, the value of MCC was 0.33, and the values of SE and SP were 75.76% and 76.00%, respectively. In Wu’s work, the machine learning model based on structural alerts and daily dose as input features showed a balanced accuracy of 69%, MCC of 0.47, and AUC of 70% on the test set. Our model covered more compounds and showed better predictive ability.

**Table 3:** Performances of best performed models on validation set.

Model	ACC (%)	SE (%)	SP (%)	MCC	AUC
MACCS_SVM	76.26	75.76	76.31	0.33	0.84
ExtendFP_SVM	79.05	57.58	81.23	0.27	0.78
KRFP_SVM	78.77	72.73	79.38	0.35	0.80
SubFP_SVM	76.54	66.67	77.54	0.29	0.81
MACCS_kNN	68.16	84.85	66.46	0.31	0.76
ExtendFP_ kNN	76.26	60.61	77.85	0.25	0.78

### The comparisons of molecular properties between DIAD and non-DIAD chemicals

**Figure 3:**
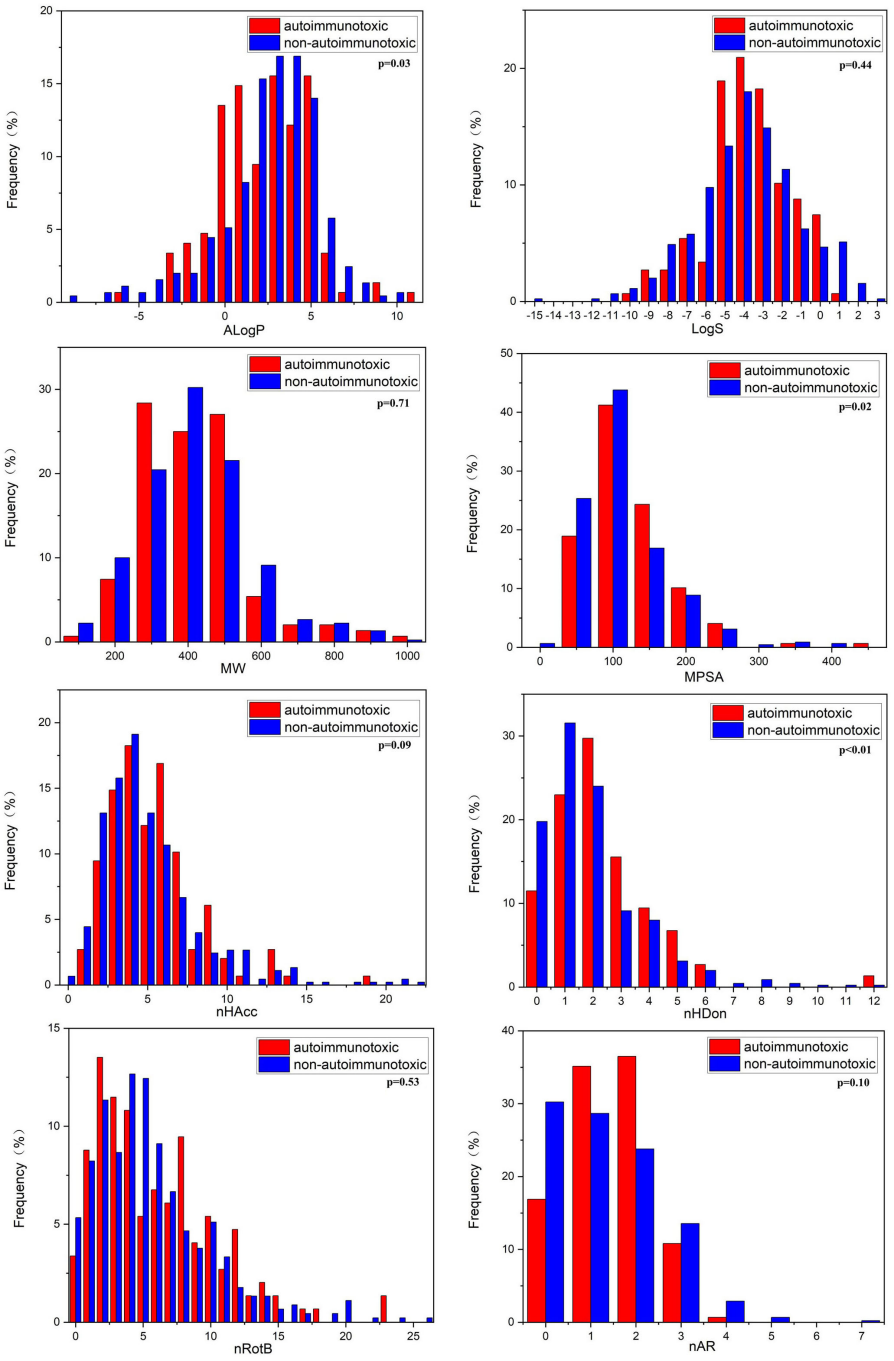
Distributions of the molecular properties for DIAD and non-DIAD chemicals.

In this study, we compared the distributions of several important molecular properties between DIAD and non-DIAD structures, as shown in **Figure 3** and **Table 4**. The results indicated that several properties were significantly different between DIAD and non-DIAD groups, including AlogP, nHDon, and MPSA. The lipophilicity of compounds is always represented with AlogP property. The median AlogP of DIAD group was 1.92 (-0.25, 3.75), which was significantly lower than that of non-DIAD group with 2.60 (0.89, 3.99), with p = 0.03. It indicated that lipophilicity should be a molecular property associated with chemical DIAD toxicity. Meanwhile, there may be no association between the molecular solubility in water with the DIAD toxicity, since there was no significant difference in logS between DIAD and non-DIAD groups (p = 0.44).

**Table 4:** Distributions of the molecular properties for DIAD and non-DIAD chemicals.

Molecular properties	M (P25, P75)		p values
	DIAD chemicals	non-DIAD chemicals	
ALogP	1.92 (-0.25, 3.75)	2.60 (0.89, 3.99)	0.03
logS	-4.15 (-5.37, -2.86)	-4.36 (-5.98, -2.74)	0.44
MW	354.92 (259.60, 429.03)	353.37 (272.14, 441.53)	0.71
MPSA	95.08 (58.29, 120.70)	78.29 (49.77, 110.51)	0.02
nHAcc	5 (3, 7)	4 (3, 6)	0.09
nHDon	2 (1, 3)	1 (1, 2)	<0.01
nRotB	5 (2, 8)	5 (3, 8)	0.53
nAR	1 (1, 2)	1 (0, 2)	0.10

The median MPSA of DIAD group (95.08 (58.29, 120.70)) was larger than that of non-DIAD group (78.29 (49.77, 110.51)), with p = 0.02, while the median MW was not significantly different between DIAD and non-DIAD groups (p = 0.71). The results suggested that DIAD chemicals may have larger polar surface area, and there may be no significant correlation between the chemical DIAD toxicity and structure size.

The analysis for chemical hydrogen bonding ability (nHAcc and nHDon) suggested that nHDon may be obviously associated with DIAD toxicity, but the nHAcc was not. The median nHDon of DIAD group was 2 (1, 3), and that of non-DIAD group was 1 (1, 2), with p < 0.01. The difference is not significant (p=0.09) in nHAcc between the groups.

The DIAD toxicity was also not obviously associated with nRotB and nAR, since the differences between DIAD and non-DIAD structures were not significant (p = 0.53 for nRotB, p = 0.10 for nAR).

In fact, the individual chemical descriptors are not sufficient to fully explain the mechanism of DIAD toxicity, since DIAD toxicity is a very complex endpoint. But we think the results of the study could provide useful information for a further understanding of DIAD toxicity.

### Structural alerts responsible for DIAD toxicity

In this study, only the fragments existed in ≥ 6 structures were kept for the structural alert detection. We identified the privileged substructures which presented much more frequently in DIAD structures than in non-DIAD structures, with f-score ≥ 0.018 and positive rate (PR) ≥ 0.75. Finally, we obtained 14 representative fragments for DIAD toxicity. More structural alerts responsible for DIAD were proposed in this study than Wu’s work. Six substructures appeared in DIAD chemicals only, which covered 32 DIAD drugs. All the privileged substructures were listed in **Table 5**.

**Table 5:** Structural alerts responsible for chemical DIAD toxicity detected from KRFP fragments.

No.	General structure	Num _P	Num _N	f-score	PR
1	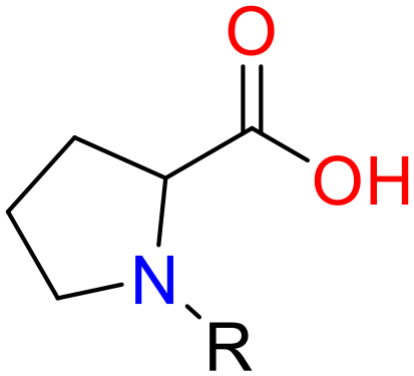	14	0	0.065	1.00
2	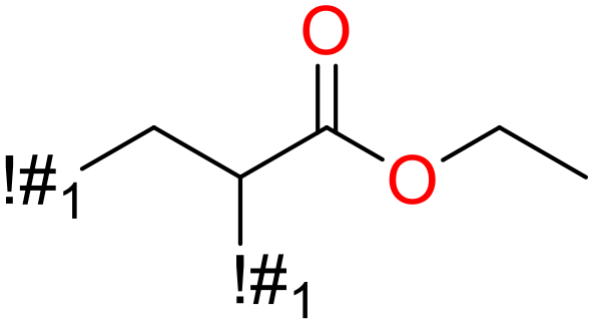	8	0	0.036	1.00
3	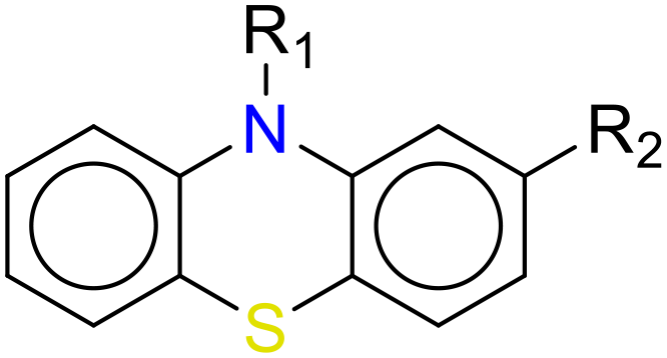	8	0	0.036	1.00
4	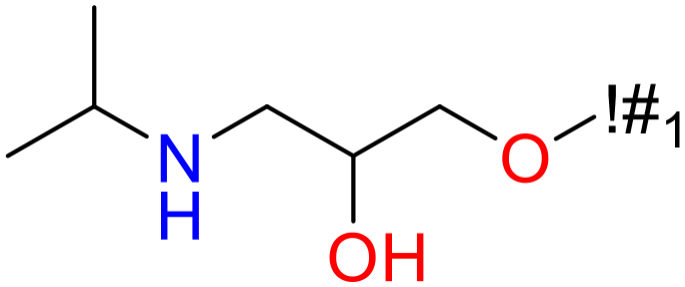	6	0	0.026	1.00
5	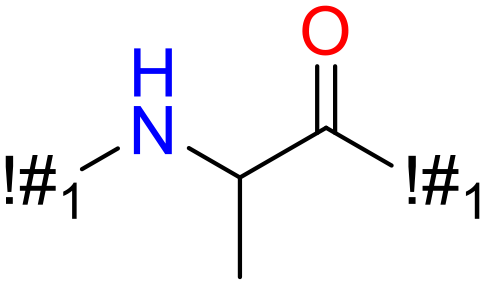	12	1	0.051	0.92
6	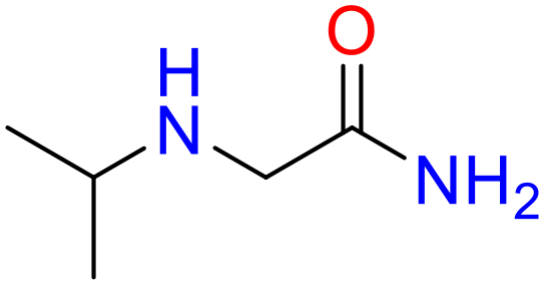	17	2	0.072	0.89
7	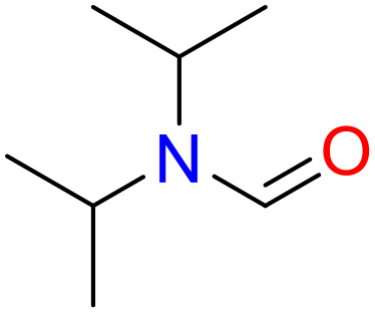	6	1	0.022	0.86
8	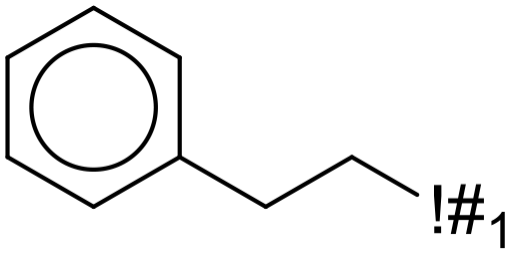	20	5	0.075	0.80
9	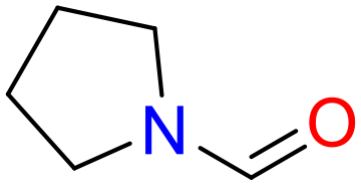	15	4	0.053	0.79
10	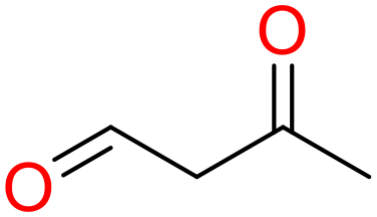	7	2	0.023	0.78
11	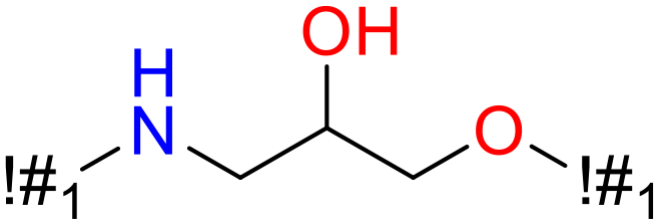	7	2	0.023	0.78
12	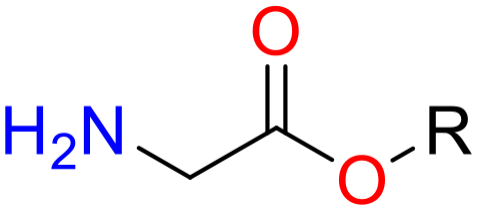	9	3	0.029	0.75
13	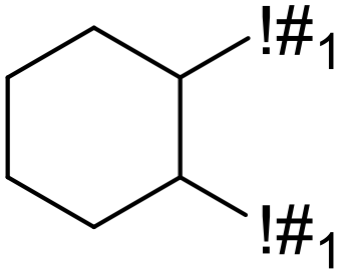	6	2	0.019	0.75
14	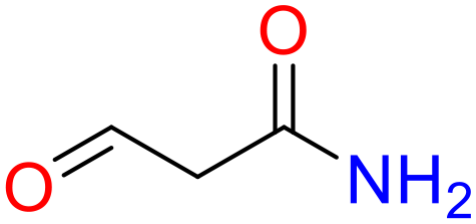	6	2	0.019	0.75

Oxidative stress is common in many autoimmune diseases and is accompanied by overproduction of reactive oxygen species (ROS) and reactive nitrogen (RNS). The role of oxidative stress in autoimmune diseases is complex and unclear. Smallwood, et al. provided insights on the pathophysiological events of oxidative stress in autoimmune rheumatic diseases (50). The role of ROS and RNS in the occurrence, detection and treatment of autoimmune diseases was summarized. In the present study, most of the structural alerts (No.1, No.2, No.4, No.5, No.6, No.7, No.9, No.10, No.11, No.12, and No.14) have the potential to produce ROS or RNS (51–56), which we infer may be related to their role in inducing autoimmune diseases. Among them, the No.1 fragments has also been identified as an alert for nephrotoxicity in our previous study. Interestingly, Hultqvist, et al. reported the protective role of ROS in autoimmune disease (57), just as many of the DIAD drugs we collected are themselves used to treat autoimmune diseases. Phenothiazine was demonstrated can induce the increase in thyroid autoantigens and costimulatory molecules on thyroid cells, which may be a pathophysiological mechanism for drug-induced autoimmunity (58). In our study, we found phenothiazine (No.3) only presented in DIAD positive structures (8 drugs).

The chemical structures used in this study only contained clinical drugs already approved on the market, and the limitation of chemical space may hinder the generalization ability of the structural alerts. Nevertheless, these privileged substructures found in this study can provide the alert help, to a certain extent, for the early assessment and mechanism of action of DIAD toxicity.

### Availability of machine learning models and structural alerts

**Figure 4:**
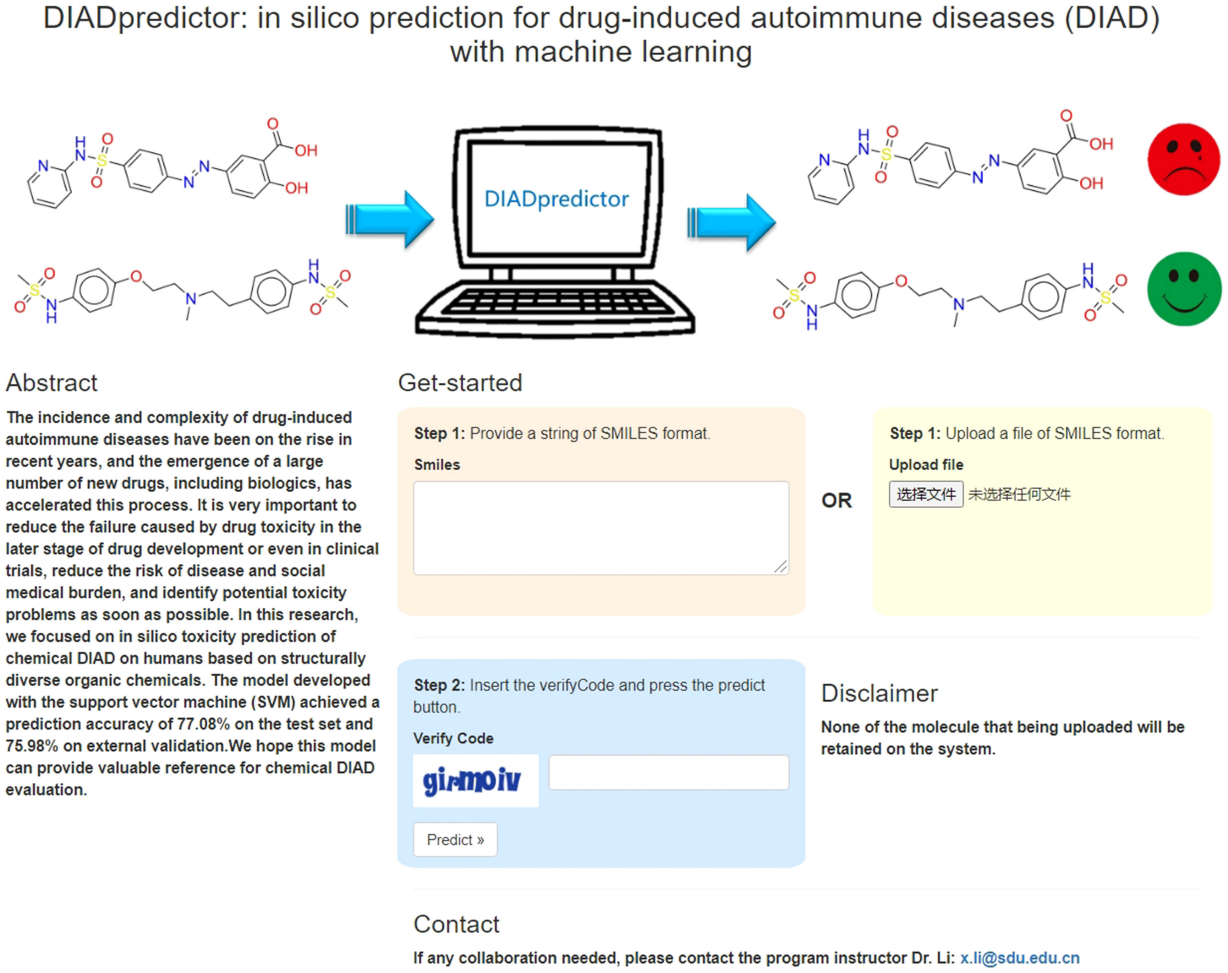
Main page of DIAD predictor web server. From this page, users can submit the query structures.

We made the best performed model developed with SVM method and MACCS keys available at a webserver named DIADpredictor, which can be freely accessed *via* http://diad.sapredictor.cn/. As shown in **Figure 4**, users can upload a.smi file or print the SMILES formula to predict whether the chemicals have DIAD toxicity freely.

The structural alerts responsible for DIAD toxicity have been integrated into SApredictor (http://www.sapredictor.cn/) (42), which is an expert system for screening chemicals against structural alerts. Users can evaluate the DIAD potential for query structures, and find the specific structural alerts which leading to DIAD toxicity intuitively. It should be noted that the web servers are not suitable for inorganics and organometallic compounds, since they were excluded from the modeling dataset.

## Conclusions

In summary, we developed the machine learning models for DIAD toxicity based on the DIAD data of clinical medications. The model developed with SVM method and MACCS keys performed best on validations. We made it available freely at http://diad.sapredictor.cn. The analysis of molecular properties for DIAD and non-DIAD compounds indicated that AlogP, molecular polar surface area (MPSA), and the number of hydrogen bond donors (nHDon) may be obviously associated with chemical DIAD toxicity. In addition, the structural alerts responsible for chemical DIAD toxicity were detected from defined fragments, and made available on SApredictor (www.sapredictor.cn). The computational models and the structural features could provide useful information and understanding for DIAD toxicity in drug and chemical hazard assessment.

## Data availability statement

The original contributions presented in the study are included in the article/**Supplementary Material**. Further inquiries can be directed to the corresponding author.

## Author contributions

HG: Data curation, Methodology, Software, Investigation, Writing - original draft. PTZ: Data curation, Methodology, Software, Investigation. RZ: Data curation, Methodology, Software, Investigation, Writing - original draft. YH: Data curation, Methodology, Validation, Visualization, Writing - review and editing. PZ: Methodology, Validation, Writing - review and editing. XC: Methodology, Validation, Writing - review and editing. XH: Methodology, Validation, Writing - review and editing. XL: Conceptualization, Project administration, Funding acquisition, Writing - review and editing. All authors contributed to the article and approved the submitted version.

## Funding

This work was supported by the National Natural Science Foundation of China (grant 81803433) and the Special Research project of Clinical Toxicology of Chinese Society of Toxicology (CST2020CT104).

## Acknowledgments

We would like to thank the staff at the Center for Big Data Research in Health and Medicine, The First Affiliated Hospital of Shandong First Medical University & Shandong Provincial Qianfoshan Hospital, for their valuable contribution. The authors also gratefully acknowledge the encouragement and support from Miss Chaoyue Yang and Miss Liying Zhao.

## Conflict of interest

The authors declare that the research was conducted in the absence of any commercial or financial relationships that could be construed as a potential conflict of interest.

## Publisher’s note

All claims expressed in this article are solely those of the authors and do not necessarily represent those of their affiliated organizations, or those of the publisher, the editors and the reviewers. Any product that may be evaluated in this article, or claim that may be made by its manufacturer, is not guaranteed or endorsed by the publisher.
