# Supplementary material for: Modeling and insights into the structural characteristics of drug-induced autoimmune diseases
Source: Front Immunol. 2022 Oct 24;13:1015409. doi: 10.3389/fimmu.2022.1015409 (PMC9637949; doi:10.3389/fimmu.2022.1015409)
Supplement: Supplementary file 1 [file DataSheet_1.pdf]

## Methodologies for structure preparation

The preparation of chemical structures was mainly carried out using pipeline pilot and OCHEM tools. First, the Pipeline Pilot was employed to build a protocol (seen in Figure 1) consisting of multiple components. Using this protocol, only the main ingredients in mixtures were kept, the inorganic and organometallic compounds were removed, and salts were recognized and converted to the parent forms. Then, we used Online Chemical Database and Modeling Environment (OCHEM) to identify and remove duplicates, since the duplicate compounds can dramatically bias performance of the methods and conclusion. The Excel tables with the structures were uploaded to OCHEM web site, selecting the created basket and clicking "Show only duplicates", and all the duplicates were displayed.

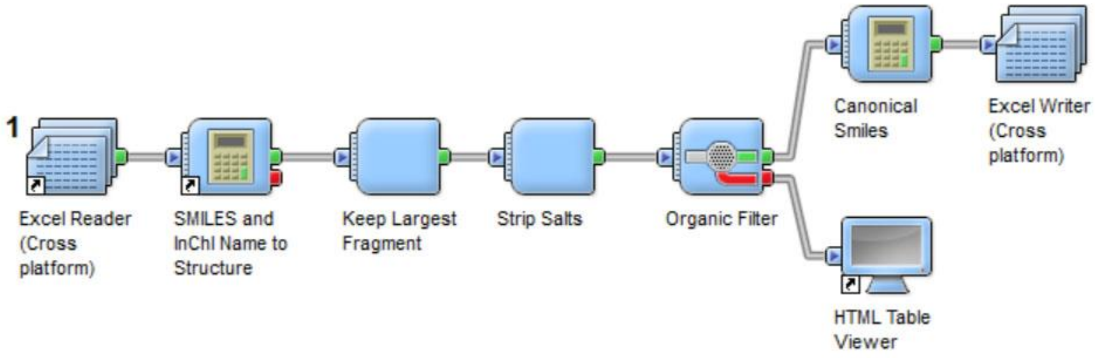


**Figure 1** Pipeline pilot protocol for structure preparation

**Table S1 Chemical structures used for modeling and analysis**

| Label | Smiles | Dataset |
|---|---|---|
| 1 | CCC1(C(=O)NCNC1=O)c2ccccc2 | training |
| 1 | CC(CN1c2ccccc2Sc3ccccc13)N(C)C | training |
| 1 | CC(=O)O[C@H]1Cc2ccccc2N(C(=O)N)c3ccccc13 | training |
| 1 | [O-][N+](=O)c1oc(\C=N\N2CC(=O)NC2=O)cc1 | training |
| 1 | CCCCCCCCCC(=O)OCCN1CCN(CCCN2c3ccccc3Sc4ccc(cc24)C(F)(F)F)CC1 | training |
| 1 | CC[C@H]1OC(=O)[C@H](C)[C@@H](O[C@H]2C[C@@](C)(OC)[C@@H](O)[C@H](C)O2)[C@H](C)[C@@H](O[C@@H]3O[C@H](C)C[C@@H]([C@H]3O)N(C)C)[C@](C)(O)C[C@@H](C)C(=O)[C@H](C)[C@@H](O)[C@]1(C)O | training |
| 1 | CN1C(=C([O-])c2ccccc2S1(=O)=O)C(=O)Nc3ncc(C)s3 | training |
| 1 | Cc1c(OCC(F)(F)F)ccnc1CS(=O)c2nc3ccccc3[nH]2 | training |
| 1 | CC1(C)S[C@@H]2[C@H](NC(=O)[C@H](N)c3ccc(O)cc3)C(=O)N2[C@H]1C(=O)O | training |
| 1 | CCCC(=O)Nc1ccc(OCC(O)CNC(C)C)c(c1)C(=O)C | training |
| 1 | CCC1(C)CC(=O)NC1=O | training |
| 1 | COCCCOc1ccnc(CS(=O)c2nc3ccccc3[nH]2)c1C | training |
| 1 | Nc1nnc(c(N)n1)c2cccc(Cl)c2Cl | training |
| 1 | CN1C(=O)C(=C(N=C1C(C)(C)NC(=O)c2oc(C)nn2)C(=O)NCc3ccc(F)cc3)[O-] | training |
| 1 | CC(C)NCC(O)COc1cccc2[nH]ccc12 | training |
| 1 | CCCNCC(O)COc1ccccc1C(=O)CCc2ccccc2 | training |
| 1 | CC(=O)OC1(CCC2C3C=C(Cl)C4=CC(=O)C5CC5C4(C)C3CCC12C)C(=O)C | training |
| 1 | C[C@@H]1[C@H]2[C@H]([O-])[C@H]3[C@H](N(C)C)C(=O)C(=C([O-])[C@@]3(O)C(=O)C2=C([O-])c4c([O-])cccc14)C(=O)N | training |
| 1 | OC(=O)[C@@H]1C[C@H](CN1C(=O)CP(=O)(O)CCCCc2ccccc2)C3CCCCC3 | training |
| 1 | Cc1cc(cc(C)c1Oc2nc(Nc3ccc(cc3)C#N)nc(N)c2Br)C#N | training |
| 1 | CC[C@H](C)C(=O)O[C@H]1C[C@@H](C)C=C2C=C[C@H](C)[C@H](CC[C@@H]3C[C@@H](O)CC(=O)O3)[C@@H]12 | training |
| 1 | CC(C)(C)NC(=O)[C@@H]1CC2CCCCC2CN1C[C@@H](O)[C@H](Cc3ccccc3)NC(=O)[C@H](CC(=O)N)NC(=O)c4ccc5ccccc5n4 | training |
| 1 | NC(=O)N1c2ccccc2C=Cc3ccccc13 | training |
| 1 | Cc1cc2OCOc2cc1CC(=O)c3sccc3S(=O)(=O)Nc4onc(C)c4Cl | training |
| 1 | CC(C)CCCC(C)CCCC(C)CCCC(=CCC1=C(C)C(=O)c2ccccc2C1=O)C | training |
| 1 | C[NH+](C)C1C2CC3C(=C([O-])c4c(O)cccc4C3(C)O)C(=O)C2(O)C(=O)C(=C(N)[O-])C1=O | training |
| 1 | NS(=O)(=O)Cc1noc2ccccc12 | training |
| 1 | N[C@@H](Cc1ccc(O)cc1)C(=O)O | training |
| 1 | Cc1onc(NS(=O)(=O)c2ccc(N)cc2)c1 | training |
| 1 | CN1CCN(CCCN2c3ccccc3Sc4ccc(cc24)C(F)(F)F)CC1 | training |
| 1 | NC(=O)N1c2ccccc2CC(=O)c3ccccc13 | training |
| 1 | [O-]C(=O)Cc1ccccc1Nc2c(Cl)cccc2Cl | training |
| 1 | CC(C)c1c(C(=O)Nc2ccccc2)c(c3ccccc3)c(c4ccc(F)cc4)n1CC[C@@H](O)C[C@@H](O)CC(=O)O | training |
| 1 | CCOC(=O)C(CCc1ccccc1)NC(C)C(=O)N2C3CCCC3CC2C(=O)O | training |
| 1 | CCNC1CC(C)S(=O)(=O)c2sc(cc12)S(=O)(=O)N | training |
| 1 | COC(=O)N[C@H](C(=O)N[C@@H](Cc1ccccc1)[C@@H](O)CN(Cc2ccc(cc2)c3ccccn3)NC(=O)[C@@H](NC(=O)OC)C(C)(C)C)C(C)(C)C | training |
| 1 | C[C@H](N[C@@H](CCc1ccccc1)C(=O)O)C(=O)N2Cc3ccccc3C[C@H]2C(=O)O | training |
| 1 | Cc1ccnc2N(C3CC3)c4ncccc4C(=O)Nc12 | training |
| 1 | Nc1nc2ccc(OC(F)(F)F)cc2s1 | training |
| 1 | Nc1cccc2C(=O)N(Cc12)C3CCC(=O)NC3=O | training |
| 1 | CCOC(=O)C(CCc1ccccc1)NC(C)C(=O)N2CCCC2C(=O)O | training |

| | | |
|---|---|---|
| 1 | COCc1c(nc(C(C)C)c(C=CC(O)CC(O)CC(=O)O)c1c2ccc(F)cc2)C(C)C | training |
| 1 | CN1CCN(CC1)C2=Nc3cc(Cl)ccc3Nc4ccccc24 | training |
| 1 | OC(=O)Cc1ccccc1Nc2c(Cl)cccc2Cl | training |
| 1 | Nc1nc(NC2CC2)c3ncn(C4CC(CO)C=C4)c3n1 | training |
| 1 | C[C@@](Cc1ccc(O)c(O)c1)(NN)C(=O)O | training |
| 1 | CC(=O)OC1C(=O)C2(C)C(O)CC3OCC3(OC(=O)C)C2C(OC(=O)c4ccccc4)C5(O)CC(OC(=O)C(O)C(NC(=O)c6ccccc6)c7ccccc7)C(=C1C5(C)C)C | training |
| 1 | CCC[C@H](N[C@H](C)C(=O)N1[C@H]2CCCC[C@H]2C[C@H]1C(=O)O)C(=O)OCC | training |
| 1 | Cc1cccc(Nc2ccccc2C(=O)O)c1C | training |
| 1 | NNc1nncc2ccccc12 | training |
| 1 | CCC(C)C(=O)OC1CC(O)C=C2C=CC(C)C(CCC(O)CC(O)CC(=O)O)C12 | training |
| 1 | NCCCC[C@H](N[C@@H](CCc1ccccc1)C(=O)O)C(=O)N2CCC[C@H]2C(=O)O | training |
| 1 | OCCN1CCN(CCCN2c3ccccc3Sc4ccc(cc24)C(F)(F)F)CC1 | training |
| 1 | CC(=C(C#N)C(=O)Nc1ccc(cc1)C(F)(F)F)O | training |
| 1 | CN1CCN(CCCN2c3ccccc3Sc4ccc(Cl)cc24)CC1 | training |
| 1 | Nc1nc(F)nc2c1ncn2C3OC(CO)C(O)C3O | training |
| 1 | CC(C)N(CCC(C(=O)N)(c1ccccc1)c2ccccn2)C(C)C | training |
| 1 | CCC(C)C1NC(=O)C(Cc2ccc(O)cc2)NC(=O)C(N)CSSCC(NC(=O)C(CC(=O)N)NC(=O)C(NC1=O)C(C)O)C(=O)N3CCCC3C(=O)NC(CC(C)C)C(=O)NCC(=O)N | training |
| 1 | NC1=NC(=O)N(C=C1)C2CSC(CO)O2 | training |
| 1 | [NH-]C1CCCCC1[NH-] | training |
| 1 | CCCC1=CC(=O)NC(=S)N1 | training |
| 1 | C[C@@H]1C2C(O)C3[C@H](N(C)C)C(=O)C(=C(O)[C@@]3(O)C(=O)C2=C(O)c4c(O)cccc14)C(=O)N | training |
| 1 | C[C@@H](N[C@@H](CCc1ccccc1)C(=O)O)C(=O)N2CCC[C@H]2C(=O)O | training |
| 1 | CN[C@H]1[C@H](O)[C@@H](O)[C@H](CO)O[C@H]1O[C@H]2[C@H](O[C@H]3[C@H](O)[C@@H](O)[C@H](N=C(N)N)[C@@H](O)[C@@H]3N=C(N)N)O[C@@H](C)[C@]2(O)C=O | training |
| 1 | CC(=O)OC12COC1CC(O)C3(C)C2C(OC(=O)c4ccccc4)C5(O)CC(OC(=O)C(O)C(NC(=O)OC(C)(C)C)c6ccccc6)C(=C(C(O)C3=O)C5(C)C)C | training |
| 1 | CCN1C(=O)NC(C1=O)c2ccccc2 | training |
| 1 | CSc1ccc2Sc3ccccc3N(CCC4CCCCN4C)c2c1 | training |
| 1 | CCC(C)(C)C(=O)O[C@H]1C[C@@H](C)C=C2C=C[C@H](C)[C@H](CC[C@@H]3C[C@@H](O)CC(=O)O3)[C@@H]12 | training |
| 1 | OC(=O)CN1C(=O)[C@H](CCc2ccccc12)N[C@H](CCc3ccccc3)C(=O)O | training |
| 1 | CN1C(=O)CC(=O)N(c2ccccc2)c3cc(Cl)ccc13 | training |
| 1 | C[C@H](N[C@@H](CCc1ccccc1)C(=O)O)C(=O)N2[C@H]3CCC[C@H]3C[C@H]2C(=O)O | training |
| 1 | COc1cc(\C=C/2\SC(=O)N(CC(=O)Nc3ccc(F)cc3F)C2=O)ccc1Oc4ccc(cc4[N+](=O)[O-])[N+](=O)[O-] | training |
| 1 | CCC(C)C(=O)OC1CC(C)C=C2C=CC(C)C(CCC3CC(O)CC(=O)O3)C12 | training |
| 1 | CN(C)C1C2C(O)C3C(=C(O)c4c(O)cccc4C3(C)O)C(=O)C2(O)C(=C(C(=O)N)C1=O)[O-] | training |
| 1 | CC(C)NCC(O)COc1ccc(CC(=O)N)cc1 | training |
| 1 | CN(C)CCCN1c2ccccc2CCc3ccc(Cl)cc13 | training |
| 1 | CC1CN(CC(C)N1)c2c(F)c(N)c3C(=O)C(=CN(C4CC4)c3c2F)C(=O)O | training |
| 1 | CC1Cc2ccccc2N1NC(=O)c3ccc(Cl)c(c3)S(=O)(=O)N | training |
| 1 | COc1cc2N(C=C(C(=O)O)C(=O)c2cc1Cc3cccc(Cl)c3F)[C@H](CO)C(C)C | training |
| 1 | CCC(=O)OC(OP(=O)(CCCCc1ccccc1)CC(=O)N2CC(CC2C(=O)[O-])C3CCCCC3)C(C)C | training |
| 1 | NC1CC1c2ccccc2 | training |
| 1 | CC(NC(CCc1ccccc1)C(=O)O)C(=O)N2C3CCCCC3CC2C(=O)O | training |
| 1 | COc1ccc2nccc([C@H](O)[C@H]3C[C@@H]4CCN3C[C@@H]4C=C)c2c1 | training |
| 1 | CC(C)NCC(O)COc1cccc2ccccc12 | training |

| | | |
|---|---|---|
| 1 | CC(C)(C)NC(=O)C1CN(Cc2cccnc2)CCN1CC(O)CC(Cc3ccccc3)C(=O)NC4C(O)Cc5ccccc45 | training |
| 1 | O[C@@H](CC[C@@H]1[C@H](N(C1=O)c2ccc(F)cc2)c3ccc(O)cc3)c4ccc(F)cc4 | training |
| 1 | CC(C)CC(N(C)C)C1(CCC1)c2ccc(Cl)cc2 | training |
| 1 | CC[C@H](C)C(=O)O[C@H]1C[C@H](O)C=C2C=C[C@H](C)[C@H](CC[C@@H](O)C[C@@H](O)CC(=O)O)[C@@H]12 | training |
| 1 | CCOC(=O)C(CCc1ccccc1)NC2CCc3ccccc3N(CC(=O)O)C2=O | training |
| 1 | C[C@H](CS)C(=O)N1CCC[C@H]1C(=O)O | training |
| 1 | CC1C2C(O)C3C([NH+](C)C)C(=O)C(=C(N)[O-])C(=O)C3(O)C(=O)C2=C([O-])c4c(O)cccc14 | training |
| 1 | CCCNC1CCc2nc(N)sc2C1 | training |
| 1 | CC(C)CN(C[C@@H](OP(=O)([O-])[O-])[C@H](Cc1ccccc1)NC(=O)O[C@H]2CCOC2)S(=O)(=O)c3ccc(N)cc3 | training |
| 1 | O=C1NC=Nc2[nH]ncc12 | training |
| 1 | NNC(=O)c1ccncc1 | training |
| 1 | CCOC(=O)C(CCc1ccccc1)NC(C)C(=O)N2Cc3ccccc3CC2C(=O)O | training |
| 1 | O=C1NC(=O)C(N1)(c2ccccc2)c3ccccc3 | training |
| 1 | Nc1ccc(O)c(c1)C(=O)O | training |
| 1 | COc1cc2C[C@H](N(Cc2cc1OC)C(=O)[C@H](C)N[C@@H](CCc3ccccc3)C(=O)O)C(=O)O | training |
| 1 | COc1cc(C)c(C=CC(=CC=CC(=CC(=O)O)C)C)c(C)c1C | training |
| 1 | CCCCCCCCCCCC(CC1OC(=O)C1CCCCCC)OC(=O)C(CC(C)C)NC=O | training |
| 1 | CN(C)CCCC1(OCc2cc(ccc12)C#N)c3ccc(F)cc3 | training |
| 1 | CCOC(=O)C(CCc1ccccc1)NC2CCCN3CCCC(N3C2=O)C(=O)O | training |
| 1 | Cc1cc(C=CC#N)cc(C)c1Nc2ccnc(Nc3ccc(cc3)C#N)n2 | training |
| 1 | CC(C)NCC(O)COc1ccc(COCCOC(C)C)cc1 | training |
| 1 | CC(=O)NS(=O)(=O)c1ccc(N)cc1 | training |
| 1 | FC(F)(F)C1(OC(=O)Nc2ccc(Cl)cc12)C#CC3CC3 | training |
| 1 | Nc1nc(F)nc2c1ncn2C3OC(COP(=O)(O)O)C(O)C3O | training |
| 1 | Nc1nc(Cl)nc2c1ncn2C3CC(O)C(CO)O3 | training |
| 1 | C[NH+](C)C1C2CC3C(O)c4c(Cl)ccc(O)c4C(=C3C(=O)C2(O)C(=O)C(=C(N)[O-])C1=O)O | training |
| 1 | CCCC(CCC)C(=O)O | training |
| 1 | C[C@](N)(Cc1ccc(O)c(O)c1)C(=O)O | training |
| 1 | NC(=O)NO | training |
| 1 | CC(=O)N(O)CCCCCNC(=O)CCC(=O)N(O)CCCCCNC(=O)CCC(=O)N(O)CCCCCN | training |
| 1 | Clc1cccc(N2CCN(CCCCOc3ccc4CCC(=O)Nc4c3)CC2)c1Cl | training |
| 0 | CO[C@H]1C=CO[C@@]2(C)Oc3c(C)c(O)c4c(O)c(NC(=O)C(=CC=C[C@H](C)[C@H](O)[C@@H](C)[C@@H](O)[C@@H](C)[C@H](OC(=O)C)[C@@H]1C)C)c5c(nc6cc(C)ccn56)c4c3C2=O | training |
| 0 | O=C1[C@@H]2[C@H]3CC[C@H](C3)[C@@H]2C(=O)N1C[C@@H]4CCCC[C@H]4CN5CCN(CC5)c6nsc7ccccc67 | training |
| 0 | CC(C)(Oc1ccc(cc1)C(=O)c2ccc(Cl)cc2)C(=O)O | training |
| 0 | OCCN1CC(O)C(O)C(O)C1CO | training |
| 0 | C[C@]12CCC3[C@@H](CC[C@H]4NC(=O)C=C[C@]34C)[C@@H]1CC[C@@H]2C(=O)Nc5cc(ccc5C(F)(F)F)C(F)(F)F | training |
| 0 | CNC1C(O)C(O)C(CO)OC1OC2C(OC3C(O)C(O)C(N=C(N)N)C(O)C3N=C(N)N)OC(C)C2(O)C=O | training |
| 0 | CC(C)OC(=O)[C@H](C)NP(=O)(OC[C@H]1O[C@@H](N2C=CC(=O)NC2=O)C(C)(F)[C@@H]1O)Oc3ccccc3 | training |
| 0 | NC1=NCC2N1c3ccccc3Cc4ccccc24 | training |
| 0 | NCCS | training |
| 0 | CC(C)OC(=O)C(C)(C)Oc1ccc(cc1)C(=O)c2ccc(Cl)cc2 | training |
| 0 | CCOCCP(CCOCC)CCP(CCOCC)CCOCC | training |
| 0 | COc1ccc(CCO[C@@H]2CCCC[C@H]2N3CC[C@@H](O)C3)cc1OC | training |
| 0 | CCN(CC)CCNC(=O)c1cc(Cl)c(N)cc1OC | training |

| | | |
|---|---|---|
| 0 | CO[C@]12[C@H]3N[C@H]3CN1C4=C([C@H]2COC(=O)N)C(=O)C(=C(C)C4=O)N | training |
| 0 | OC(C(=O)O[C@@H]1C[N+]2(CCCOc3ccccc3)CCC1CC2)(c4cccs4)c5cccs5 | training |
| 0 | NC(=NC(=NCCCCCCN=C(N)N=C(N)Nc1ccc(Cl)cc1)N)Nc2ccc(Cl)cc2 | training |
| 0 | CN(C)C(=O)C(CC[NH+]1CCC(O)(CC1)c2ccc(Cl)cc2)(c3ccccc3)c4ccccc4 | training |
| 0 | CCC(C(C)CN(C)C)c1cccc(O)c1 | training |
| 0 | NC1=NC(=O)N(C[C@@H](CO)OCP(=O)(O)O)C=C1 | training |
| 0 | CCN[C@H]1CN(CCCOC)S(=O)(=O)c2sc(cc12)S(=O)(=O)N | training |
| 0 | CS(=O)(=O)c1ccc(cc1)C2=C(C(=O)OC2)c3ccccc3 | training |
| 0 | CC(=O)Oc1ccccc1C(=O)Nc2ncc(s2)[N+](=O)[O-] | training |
| 0 | COc1cc(cc(OC)c1OC)C2C3C(COC3=O)C(O)c4cc5OCOc5cc24 | training |
| 0 | COc1nc2ccc(Br)cc2cc1[C@H](c3ccccc3)[C@@](O)(CCN(C)C)c4cccc5ccccc45 | training |
| 0 | OC[C@H]1O[C@H]([C@H](O)[C@@H]1OP(=O)(O)OC[C@H]2O[C@H]([C@H](O)[C@@H]2O)N3C=CC(=O)NC3=O)N4C=CC(=O)NC4=O | training |
| 0 | NCc1ccc(cc1)S(=O)(=O)N | training |
| 0 | COC(=O)C1=C(C)NC(=C(C1c2cccc(c2)[N+](=O)[O-])C(=O)OCC[NH+](C)Cc3ccccc3)C | training |
| 0 | CCCCCc1cc(O)c2C3C=C(C)CCC3C(C)(C)Oc2c1 | training |
| 0 | CN1C(=O)N(C)c2ncn(C)c2C1=O | training |
| 0 | CCCCCC(O)C=CC1C(O)CC(=O)C1CCCCCCC(=O)O | training |
| 0 | CC(CN1CC(=O)NC(=O)C1)N2CC(=O)NC(=O)C2 | training |
| 0 | C[C@@H](NCCCc1cccc(c1)C(F)(F)F)c2cccc3ccccc23 | training |
| 0 | CN(C)CCOc1ccc(cc1)C(=C(CCCl)c2ccccc2)c3ccccc3 | training |
| 0 | CCc1ccc(cc1)C(C)CCc2ccc(cc2)[N+](C)(C)C | training |
| 0 | CN1C2CCC1CC(C2)OC(=O)c3c[nH]c4ccccc34 | training |
| 0 | CN1C(=O)CN=C(c2ccccc2F)c3cc(ccc13)[N+](=O)[O-] | training |
| 0 | CCCCC1=NC2(CCCC2)C(=O)N1Cc3ccc(cc3)c4ccccc4c5nn[nH]n5 | training |
| 0 | COCCOC(=O)C1=C(C)NC(=C(C1c2cccc(c2)[N+](=O)[O-])C(=O)OC(C)C)C | training |
| 0 | CCCCCC(O)C=CC1C(O)CC(=O)C1CC=CCCCC(=O)O | training |
| 0 | CC(C)COc1ccc(cc1C#N)c2nc(C)c(s2)C(=O)O | training |
| 0 | OP(=O)(O)[O-] | training |
| 0 | CN1C2CCC1CC(C2)OC(=O)C(CO)c3ccccc3 | training |
| 0 | CCCCCCCCCCCCCCCCCCCCCCO | training |
| 0 | OC[C@@H](O)[C@@H](O)[C@H](O)[C@@H](O)C(=O)O | training |
| 0 | CCN1N=NN(CCN2CCC(COC)(CC2)N(C(=O)CC)c3ccccc3)C1=O | training |
| 0 | N[C@H](C(=O)N1[C@@H](C[C@@H]2C[C@H]12)C#N)C34C[C@H]5C[C@H](CC(O)(C5)C3)C4 | training |
| 0 | CCCSc1nc(N[C@@H]2C[C@H]2c3ccc(F)c(F)c3)c4nnn([C@@H]5C[C@H](OCCO)[C@@H](O)[C@H]5O)c4n1 | training |
| 0 | CCCc1nc(c(C(=O)O)n1Cc2ccc(cc2)c3ccccc3c4nn[nH]n4)C(C)(C)O | training |
| 0 | CC(=O)O[C@H]1[C@H](CC2C3CCC4C[C@H](O)[C@H](C[C@]4(C)C3CC[C@]12C)N5CCOCC5)[N+]6(CC=C)CCCC6 | training |
| 0 | Cn1nnc(n1)c2ccc(cn2)c3ccc(cc3F)N4C[C@H](CO)OC4=O | training |
| 0 | CCOC(=O)C1=C(COCCN)NC(=C(C1c2ccccc2Cl)C(=O)OC)C | training |
| 0 | CCN(CC)CC#CCOC(=O)C(O)(C1CCCCC1)c2ccccc2 | training |
| 0 | CC(N)Cc1ccccc1 | training |
| 0 | Clc1ccc(CON=C(Cn2ccnc2)c3ccc(Cl)cc3Cl)c(Cl)c1 | training |
| 0 | CNC(=C[N+](=O)[O-])NCCSCc1oc(CN(C)C)cc1 | training |
| 0 | CN(CC=Cc1ccccc1)Cc2cccc3ccccc23 | training |
| 0 | Clc1ccc2c(c1)C(=NCc3nncn23)c4ccccc4 | training |
| 0 | Nc1cc(Cl)c(NC2=NCCN2)c(Cl)c1 | training |
| 0 | CC(=O)OCC(=O)C12OC(C)(C)OC1CC3C4CC(F)C5=CC(=O)C=CC5(C)C4(F)C(O)CC23C | training |
| 0 | OCCOc1ccc(cc1)C(=C(CCCl)c2ccccc2)c3ccccc3 | training |
| 0 | Cc1ncc([N+](=O)[O-])n1CCO | training |
| 0 | CCN(CC)CCOc1ccc(cc1)C(=C(Cl)c2ccccc2)c3ccccc3 | training |
| 0 | CNc1ccc(\C=C\c2ccc(OCCOCCOCC[18F])nc2)cc1 | training |

| | | |
|---|---|---|
| 0 | O=C(C1CCCCC1)N2CC3N(CCc4ccccc34)C(=O)C2 | training |
| 0 | Nc1nc(cs1)C(=CCC(=O)O)C(=O)NC2C3SCC=C(N3C2=O)C(=O)O | training |
| 0 | C[N+](C)(C)CC(=O)[O-] | training |
| 0 | CN(C)C(=O)Oc1ccc[n+](C)c1 | training |
| 0 | COC(=O)N(C)c1c(N)nc(nc1N)c2nn(Cc3ccccc3F)c4ncccc24 | training |
| 0 | NCC(=O)O | training |
| 0 | CCCc1nc(c(C(=O)OCC2=C(C)OC(=O)O2)n1Cc3ccc(cc3)c4ccccc4c5nn[nH]n5)C(C)(C)O | training |
| 0 | CCCC1CC(N(C)C1)C(=O)NC(C(C)Cl)C2OC(SC)C(O)C(O)C2O | training |
| 0 | NC(=NC(=O)c1nc(Cl)c(N)nc1N)N | training |
| 0 | CC1(C)OC2CC3C4CC(F)C5=CC(=O)C=CC5(C)C4(F)C(O)CC3(C)C2(O1)C(=O)CO | training |
| 0 | CCCCC(C)(C)C(O)C=CC1C(O)CC(=O)C1CCCCC=CC(=O)OC | training |
| 0 | CC(C)OC(=O)CCC\C=C\CC1C(O)CC(O)C1\C=C\C(F)(F)COc2ccccc2 | training |
| 0 | C[C@H](C=C[C@H](C)C(C)(C)O)[C@H]1CC[C@H]2C(=CC=C3C[C@@H](O)C[C@H](O)C3)CCC[C@]12C | training |
| 0 | CC(C)N=C(N)N=C(N)Nc1ccc(Cl)cc1 | training |
| 0 | CNCCc1ccccn1 | training |
| 0 | COC(=O)[C@@H]1[C@H]2CCC(C[C@@H]1c3ccc(I)cc3)N2CCCF | training |
| 0 | NC1[C@H]2CN(C[C@@H]12)c3nc4N(C=C(C(=O)O)C(=O)c4cc3F)c5ccc(F)cc5F | training |
| 0 | CC(=O)[C@@]1(O)CC[C@H]2[C@@H]3C=C(C)C4=CC(=O)CC[C@]4(C)[C@H]3CC[C@]12C | training |
| 0 | NCC(=O)CCC(=O)O | training |
| 0 | COCC1=C(N2C(SC1)C(NC(=O)C(=NOC)c3csc(N)n3)C2=O)C(=O)O | training |
| 0 | CCOC(=O)N[C@@H]1CC[C@@H]2[C@@H](C1)C[C@@H]3[C@@H]([C@@H](C)OC3=O)C2\C=C\c4ccc(cn4)c5cccc(F)c5 | training |
| 0 | C[C@]12CC[C@H]3[C@@H]([C@H](CCCCCCCCCS(=O)CCCC(F)(F)C(F)(F)F)Cc4cc(O)ccc34)[C@@H]1CC[C@@H]2O | training |
| 0 | COc1cc(ccc1Cc2cn(C)c3ccc(NC(=O)OC4CCCC4)cc23)C(=O)NS(=O)(=O)c5ccccc5C | training |
| 0 | CCS(=O)(=O)CCn1c(C)ncc1[N+](=O)[O-] | training |
| 0 | FCOC(C(F)(F)F)C(F)(F)F | training |
| 0 | OC(Cc1cccnc1)(P(=O)(O)O)P(=O)(O)O | training |
| 0 | CC(C=CC(O)C1CC1)C2CCC3C(=CC=C4CC(O)CC(O)C4=C)CCCC23C | training |
| 0 | NCC1CCC(CC1)C(=O)O | training |
| 0 | CC(C)(Oc1ccc(CCNC(=O)c2ccc(Cl)cc2)cc1)C(=O)O | training |
| 0 | O=C1N(C[C@@H]2CCCc3cccc1c23)[C@@H]4CN5CCC4CC5 | training |
| 0 | CC1(C)OC2CC3C4CC(F)C5=CC(=O)C=CC5(C)C4C(O)CC3(C)C2(O1)C(=O)CO | training |
| 0 | COc1cc2CCN3CC(CC(C)C)C(=O)CC3c2cc1OC | training |
| 0 | CC(=O)C1(O)CCC2C3C=C(Cl)C4=CC(=O)C5CC5C4(C)C3CCC12C | training |
| 0 | O[C@@H]1CO[C@@H](O[C@@H]2CO[C@@H](O)[C@H](OS(=O)(=O)O)[C@H]2OS(=O)(=O)O)[C@H](OS(=O)(=O)O)[C@H]1OS(=O)(=O)O | training |
| 0 | CC1OC1P(=O)(O)O | training |
| 0 | CCCc1c2OC(=CC(=O)c2cc3C(=O)C=C(N(CC)c13)C(=O)O)C(=O)O | training |
| 0 | Nc1nc(CC(=O)Nc2ccc(CCNC[C@H](O)c3ccccc3)cc2)cs1 | training |
| 0 | Cc1nnc(SCC2=C(N3C(SC2)C(NC(=O)Cn4cnnn4)C3=O)C(=O)[O-])s1 | training |
| 0 | NCC1OC(OC2C(N)CC(N)C(OC3OC(CO)C(O)C(N)C3O)C2O)C(N)CC1O | training |
| 0 | S=C1N=CNc2nc[nH]c12 | training |
| 0 | CN1CCC23C4CCC(=O)C2Oc5c(O)ccc(CC14)c35 | training |
| 0 | CN1CCC23C4Oc5c(O)ccc(CC1C2(O)CCC4=O)c35 | training |
| 0 | OC(COc1cccc2OC(=CC(=O)c12)C(=O)[O-])COc3cccc4OC(=CC(=O)c34)C(=O)[O-] | training |
| 0 | CN(C)c1ccc(cc1)C2CC3(C)C(CCC3(O)C(=O)C)C4CCC5=CC(=O)CCC5=C2 | training |
| 0 | CC12CCC3C(CCc4cc(OS(=O)(=O)O)ccc34)C1CCC2=O | training |

| | | |
|---|---|---|
| 0 | CC[C@H]1OC(=O)[C@H](C)C(=O)[C@H](C)[C@@H](O[C@@H]2O[C@H](C)C[C@@H]([C@H]2O)N(C)C)[C@@](C)(C[C@@H](C)C(=O)[C@@H](C)[C@H]3N(CCCCn4cnc(c4)c5cccnc5)C(=O)O[C@]13C)OC | training |
| 0 | Cc1ccc(Sc2ccccc2N3CCNCC3)c(C)c1 | training |
| 0 | COc1ccc2CC3N(C)CCC45C(Oc1c24)C(=O)CCC35O | training |
| 0 | CN[C@@H]1CCc2[nH]c3ccc(cc3c2C1)C(=O)N | training |
| 0 | CN(CCOc1ccc(NS(=O)(=O)C)cc1)CCc2ccc(NS(=O)(=O)C)cc2 | training |
| 0 | CCC1CCC[C@H](O[C@H]2CCC([C@@H](C)O2)N(C)C)C(C)C(=O)C3=C[C@H]4[C@@H]5C[C@@H](C[C@H]5C(=C[C@H]4[C@@H]3CC(=O)O1)C)O[C@@H]6O[C@@H](C)[C@H](OC)[C@@H](OC)[C@H]6OC | training |
| 0 | CC(C)C(=O)OCC(=O)[C@@]12OC(O[C@@H]1C[C@H]3[C@@H]4CCC5=CC(=O)C=C[C@]5(C)[C@H]4[C@@H](O)C[C@]23C)C6CCCCC6 | training |
| 0 | C[N+]1(CC2CC2)CC[C@]34[C@H]5Oc6c(O)ccc(C[C@@H]1[C@]3(O)CCC5=O)c46 | training |
| 0 | C[C@@H](c1cnc[nH]1)c2cccc(C)c2C | training |
| 0 | CCOC(=O)c1ncn2c1CN(C)C(=O)c3cc(F)ccc23 | training |
| 0 | CCCCC[C@](C)(O)C=C[C@H]1[C@H](O)C[C@H](O)[C@@H]1CC=CCCCC(=O)O | training |
| 0 | CCN1CCCC1CNC(=O)c2cc(c(N)cc2OC)S(=O)(=O)CC | training |
| 0 | COc1ccc2CC3C4CCC(O)C5Oc1c2C45CCN3C | training |
| 0 | CCCCC(=O)O[C@@]1([C@@H](C)C[C@H]2[C@@H]3CCC4=CC(=O)C=C[C@]4(C)[C@@]3(F)[C@@H](O)C[C@]12C)C(=O)CO | training |
| 0 | COc1cc(cc(OC)c1O)c2[o+]c3cc(O)cc(O)c3cc2O | training |
| 0 | OC[C@H]1O[C@H]([C@H](O)[C@@H](O)[C@@H]1O)c2ccc(Cl)c(Cc3ccc(O[C@H]4CCOC4)cc3)c2 | training |
| 0 | CCNC(=O)CCCC=CC[C@H]1[C@@H](O)C[C@@H](O)[C@@H]1C=C[C@@H](O)CCc2ccccc2 | training |
| 1 | CN(C)CCCN1c2ccccc2Sc3ccc(Cl)cc13 | validation |
| 1 | CN(C)[C@H]1[C@@H]2C[C@@H]3Cc4c(ccc(O)c4C(=C3C(=O)[C@]2(O)C(=C(C(=O)N)C1=O)O)O)N(C)C | validation |
| 1 | CC(Cc1ccc(O)c(O)c1)(NN)C(=O)O | validation |
| 1 | NC(=O)c1ncn(n1)C2OC(CO)C(O)C2O | validation |
| 1 | CCCN(CCC)CCc1cccc2NC(=O)Cc12 | validation |
| 1 | COc1ccc(CC(C)NCC(O)c2ccc(O)c(NC=O)c2)cc1 | validation |
| 1 | CCN(CC)C(=O)\C(=C\c1cc(O)c(O)c(c1)[N+](=O)[O-])\C#N | validation |
| 1 | COc1ccc(cc1)C(CN(C)C)C2(O)CCCCC2 | validation |
| 1 | CC(C)n1c(C=CC(O)CC(O)CC(=O)O)c(c2ccc(F)cc2)c3ccccc13 | validation |
| 1 | NCCCCC(NC(CCc1ccccc1)C(=O)O)C(=O)N2CCCC2C(=O)O | validation |
| 1 | CN1CCC[C@@H]1Cc2c[nH]c3ccc(CCS(=O)(=O)c4ccccc4)cc23 | validation |
| 1 | CC1=CN(C2OC(CO)C=C2)C(=O)NC1=O | validation |
| 1 | COCCc1ccc(OCC(O)CNC(C)C)cc1 | validation |
| 1 | COc1cc(Cc2cnc(N)nc2N)cc(OC)c1OC | validation |
| 1 | Clc1ccccc1CN2CCc3sccc3C2 | validation |
| 1 | COc1ccc2nccc([C@@H](O)[C@@H]3C[C@@H]4CCN3C[C@@H]4C=C)c2c1 | validation |
| 1 | CCC[C@H](N[C@@H](C)C(=O)N1[C@H]2CCCC[C@H]2C[C@H]1C(=O)O)C(=O)O | validation |
| 1 | CC(C)CN(C[C@@H](O)[C@H](Cc1ccccc1)NC(=O)O[C@H]2CO[C@H]3OCC[C@@H]23)S(=O)(=O)c4ccc(N)cc4 | validation |
| 1 | NC(=O)Cc1cccc(C(=O)c2ccccc2)c1N | validation |
| 1 | CC(CCc1ccccc1)NCC(O)c2ccc(O)c(c2)C(=O)N | validation |
| 1 | NC1=NC(=O)N(C=C1F)[C@@H]2CS[C@H](CO)O2 | validation |
| 1 | CC[C@H]1OC(=O)[C@H](C)[C@@H](OC2CC(C)(OC)C(O)C(C)O2)[C@H](C)[C@@H](OC3OC(C)CC(C3O)N(C)C)[C@@](C)(C[C@@H](C)C(=O)[C@H](C)[C@@H](O)[C@]1(C)O)OC | validation |
| 1 | CC(C)n1c(\C=C\[C@@H](O)C[C@@H](O)CC(=O)O)c(c2ccc(F)cc2)c3ccccc13 | validation |
| 1 | CCOC(=O)[C@H](CCc1ccccc1)N[C@@H](C)C(=O)N2CCC[C@H]2C(=O)O | validation |

| | | |
|---|---|---|
| 1 | CC(C)(S)C(N)C(=O)O | validation |
| 1 | OC(=O)c1cc(ccc1O)N=Nc2ccc(cc2)S(=O)(=O)Nc3ccccn3 | validation |
| 1 | CCC[C@@]1(CCc2ccccc2)CC(=C([C@H](CC)c3cccc([N-]S(=O)(=O)c4ccc(cn4)C(F)(F)F)c3)C(=O)O1)[O-] | validation |
| 1 | COc1cccc(c1)C2(O)CCCCC2CN(C)C | validation |
| 1 | CC(=O)S[C@@H]1CC2=CC(=O)CC[C@]2(C)[C@H]3CC[C@@]4(C)[C@@H](CC[C@@]45CCC(=O)O5)[C@H]13 | validation |
| 1 | CCOC(=O)C(CCc1ccccc1)NC(C)C(=O)N2C3CCCCC3CC2C(=O)O | validation |
| 1 | CO\N=C(/C(=O)N[C@H]1[C@H]2SCC(=C(N2C1=O)C(=O)O)COC(=O)N)\c3occc3 | validation |
| 1 | CC1CC2C(CCC3(C)C2CCC3(OC(=O)C)C(=O)C)C4(C)CCC(=O)C=C14 | validation |
| 1 | OCCN1CCN(CCCN2c3ccccc3Sc4ccc(Cl)cc24)CC1 | validation |
| 0 | COC[C@@H](NC(=O)C)C(=O)NCc1ccccc1 | validation |
| 0 | CCCNS(=O)(=O)Nc1ncnc(OCCOc2ncc(Br)cn2)c1c3ccc(Br)cc3 | validation |
| 0 | CN1CCN2C(C1)c3ccccc3Cc4ccccc24 | validation |
| 0 | [O-]C(=O)P(=O)([O-])[O-] | validation |
| 0 | COc1ccc(cc1C23CC4CC(CC(C4)C2)C3)c5ccc6cc(ccc6c5)C(=O)O | validation |
| 0 | COC(=O)CCc1c(C)c2cc3nc(cc4[nH]c(cc5nc(cc1[nH]2)c(CCC(=O)O)c5C)C(=C4C)C=C)C6=CC=C([C@@H](C(=O)OC)[C@@]36C)C(=O)OC | validation |
| 0 | NCCCC(N)(C(F)F)C(=O)O | validation |
| 0 | CCOC(=O)Nc1ccc(NCc2ccc(F)cc2)cc1N | validation |
| 0 | CCN(C(=O)C)c1cccc(c1)c2ccnc3c(cnn23)C#N | validation |
| 0 | OCc1cc(ccc1O)C(O)CNCCCCCCOCCCCc2ccccc2 | validation |
| 0 | C[N+](C)(C)CC(O)CC(=O)O | validation |
| 0 | CN1CCc2cccc3c2C1Cc4ccc(O)c(O)c34 | validation |
| 0 | Clc1cccc(c1)N2CCN(CCCN3N=C4C=CC=CN4C3=O)CC2 | validation |
| 0 | CC(N)COc1c(C)cccc1C | validation |
| 0 | C[C@@H](O)[C@@H]1[C@H]2[C@@H](C)C(=C(N2C1=O)C(=O)O)S[C@@H]3CN[C@@H](C3)C(=O)Nc4cccc(c4)C(=O)O | validation |
| 0 | CN(C)CCC=C1c2ccccc2COc3ccccc13 | validation |
| 0 | COc1cc(cc(OC)c1OP(=O)(O)O)[C@H]2[C@@H]3[C@H](COC3=O)[C@H](OC4OC5COC(C)OC5C(O)C4O)c6cc7OCOc7cc26 | validation |
| 0 | COCCCN1CCC(CC1)NC(=O)c2cc(Cl)c(N)c3CCOc23 | validation |
| 0 | CC12CC(O)C3(F)C(CCC4=CC(=O)C=CC34C)C1CC(O)C2(O)C(=O)CO | validation |
| 0 | CCOc1cc(ccc1OC)[C@H](CS(=O)(=O)C)N2C(=O)c3cccc(NC(=O)C)c3C2=O | validation |
| 0 | Cc1c(C)c2OC(C)(COc3ccc(CC4SC(=O)NC4=O)cc3)CCc2c(C)c1O | validation |
| 0 | CC(C)OC(=O)CCCC=CC[C@H]1[C@@H](O)C[C@@H](O)[C@@H]1C=CC(O)COc2cccc(c2)C(F)(F)F | validation |
| 0 | CC1=CC(=O)N(O)C(=C1)C2CCCCC2 | validation |
| 0 | CC1=C(CC(=O)O)c2cc(F)ccc2C1=Cc3ccc(cc3)S(=O)C | validation |
| 0 | C(C1=NCCN1)c2cccc3ccccc23 | validation |
| 0 | Cc1ncc2CN=C(c3ccccc3F)c4cc(Cl)ccc4n12 | validation |
| 0 | C[C@@H]1O[C@@H]1P(=O)(O)[O-] | validation |
| 0 | COC(=O)C1=C(C)NC(=C(C1c2ccccc2[N+](=O)[O-])C(=O)OC)C | validation |
| 0 | CC1C(O)CCC2(C)C1CCC3(C)C2C(O)CC4C(=C(CCC=C(C)C)C(=O)O)C(CC34C)OC(=O)C | validation |
| 0 | CCC12CC(=C)C3C(CCC4=CC(=O)CCC34)C1CC[C@@]2(O)C#C | validation |
| 0 | CC\C=C/C\C=C/C\C=C/C\C=C/C\C=C/C\C=C/CCC(=O)O | validation |
| 0 | Oc1ccc2CC3N(CC4CCC4)CCC5(CCCCC35O)c2c1 | validation |
| 0 | CC12CCC3C(=CCc4cc(OS(=O)(=O)O)ccc34)C1CCC2=O | validation |
| 0 | NC(=O)CS(=O)C(c1ccccc1)c2ccccc2 | validation |
| 0 | Cc1ccc(cc1)C(=O)c2ccc(CC(=O)[O-])n2C | validation |
| 0 | NC(CCC(=O)N)C(=O)O | validation |
| 0 | Cc1c(nn(c2ccc(Cl)cc2Cl)c1c3ccc(Cl)cc3)C(=O)NN4CCCCC4 | validation |
| 0 | CN1C(=O)N(C)c2nc[nH]c2C1=O | validation |
| 0 | OCC(O)C(O)C(O)C(O)CO | validation |
| 0 | CCCCCCC(C)(C)c1cc(O)c2C3CC(=O)CCC3C(C)(C)Oc2c1 | validation |

| | | |
|---|---|---|
| 0 | C[C@@H](O)[C@@H]1[C@H]2[C@@H](C)C(=C(N2C1=O)C(=O)O)S[C@H]3CC[C@H](N3)C(=O)N(C)C | validation |
| 0 | CCCC(=O)OCOC(=O)C1=C(C)NC(=C(C1c2cccc(Cl)c2Cl)C(=O)OC)C | validation |
| 0 | Nc1ccc(cc1)S(=O)(=O)c2ccc(N)cc2 | validation |
| 0 | OC[C@H]1O[C@H](O[C@]2(CO)O[C@H](CO)[C@@H](O)[C@@H]2O)[C@H](O)[C@@H](O)[C@@H]1O | validation |
| 0 | FC(F)Oc1ccc(cc1OCC2CC2)C(=O)Nc3c(Cl)cncc3Cl | validation |
| 0 | Cc1ccc(cc1)S(=O)(=O)NC(=O)NN2CC3CCCC3C2 | validation |
| 0 | CC(N1CCC(=C)CC1)C(O)(Cn2cncn2)c3ccc(F)cc3F | validation |
| 0 | NC(=O)c1oc2ccc(cc2c1)N3CCN(CCCCc4c[nH]c5ccc(cc45)C#N)CC3 | validation |
| 0 | CNCCC=C1c2ccccc2CCc3ccccc13 | validation |
| 0 | C[C@@H]1C[C@H]2C3CCC4=CC(=O)C=C[C@]4(C)[C@@]3(Cl)[C@@H](O)C[C@]2(C)[C@@]1(O)C(=O)CCl | validation |
| 0 | CCC(=O)OC1(C(C)CC2C3CCC4=CC(=O)C=CC4(C)C3(F)C(O)CC12C)C(=O)CCl | validation |
| 0 | CC1CC2C3CCC4=CC(=O)C=CC4(C)C3(Cl)C(O)CC2(C)C1(O)C(=O)CO | validation |
| 0 | COc1cc2c(Nc3ccc(Br)cc3F)ncnc2cc1OCC4CCN(C)CC4 | validation |
| 0 | O=[N] | validation |
| 0 | CCc1cc2CC(Cc2cc1CC)NCC(O)c3ccc(O)c4NC(=O)C=Cc34 | validation |
| 0 | COc1ccc(CC2c3cc(OC)c(OC)cc3CC[N+]2(C)CCC(=O)OCCCCCOC(=O)CC[N+]4(C)CCc5cc(OC)c(OC)cc5C4Cc6ccc(OC)c(OC)c6)cc1OC | validation |
| 0 | CCc1cccc2c3CCOC(CC)(CC(=O)O)c3[nH]c12 | validation |
| 0 | OC(CC=O)C=O | validation |
| 0 | [O-]C1=NC(=O)N(C1)N=Cc2oc(cc2)c3ccc(cc3)[N+](=O)[O-] | validation |
| 0 | CC1(C)O[C@@H]2C[C@H]3C4CCC5=CC(=O)C=CC5(C)[C@H]4C(O)CC3(C)[C@@]2(O1)C(=O)CO | validation |
| 0 | CC(=O)[C@@]1(O)CC[C@H]2[C@@H]3CCC4=CC(=O)CC[C@]4(C)[C@H]3CC[C@]12C | validation |
| 0 | OC(C(=O)OC1CC2CCC(C1)[N+]23CCCC3)(c4ccccc4)c5ccccc5 | validation |
| 0 | CCCCc1ncc(C=C(Cc2cccs2)C(=O)O)n1Cc3ccc(cc3)C(=O)O | validation |
| 0 | CC(C)c1cccc(C(C)C)c1OCOP(=O)([O-])[O-] | validation |
| 0 | CC1(C)O[C@@H]2C[C@H]3[C@@H]4CCC5=CC(=O)C=C[C@]5(C)[C@@]4(F)[C@@H](O)C[C@]3(C)[C@@]2(O1)C(=O)CO | validation |
| 0 | Cc1nc2c(CCN(C(=O)c3ccc(NC(=O)c4ccccc4c5ccccc5)cc3)c6ccccc26)[nH]1 | validation |
| 0 | COc1ccc2cccc(CCNC(=O)C)c2c1 | validation |
| 0 | OC(=O)CC(O)(CC(=O)O)C(=O)O | validation |
| 0 | CC(C)(C)NCC(O)COc1cccc2CC(O)C(O)Cc12 | validation |
| 0 | CC(N)C12CC3CC(CC(C3)C1)C2 | validation |
| 0 | OC(=O)CCCN1CCC(CC1)OC(c2ccc(Cl)cc2)c3ccccn3 | validation |
| 0 | COC(=O)CCC(=O)CN | validation |
| 0 | CC1(C)OC2CC3C4CC(F)C5=CC(=O)CCC5(C)C4C(O)CC3(C)C2(O1)C(=O)CO | validation |
| 0 | OCCOC[C@H]1O[C@H](OCCO)[C@H](OCCO)[C@@H](OCCO)[C@@H]1OCCO | validation |
| 0 | CC1Nc2cc(Cl)c(cc2C(=O)N1c3ccccc3C)S(=O)(=O)N | validation |
| 0 | CS(=O)(=O)c1ccc(C(=O)Nc2ccc(Cl)c(c2)c3ccccn3)c(Cl)c1 | validation |
| 0 | COc1cc(ccc1OCCCN2CCC(CC2)c3noc4cc(F)ccc34)C(=O)C | validation |
| 0 | COCCCOc1cc(C[C@H](C[C@H](N)[C@@H](O)C[C@@H](C(C)C)C(=O)NCC(C)(C)C(=O)N)C(C)C)ccc1OC | validation |
| 0 | CN1C(=O)CN=C(c2ccccc2)c3cc(Cl)ccc13 | validation |
| 0 | NC(CCCN=C(N)N)C(=O)O | validation |
| 0 | CC(C)C(=O)Nc1ccc(c(c1)C(F)(F)F)[N+](=O)[O-] | validation |
| 0 | CCc1c(C)c2cc3nc(cc4nc(cc5[nH]c(cc1[nH]2)C(=C5C(C)O)C)C(=C4CCC(=O)O)C)c(CCC(=O)O)c3C | validation |
| 0 | Cc1c(Br)c(O)c(Br)cc1C2(OS(=O)(=O)c3ccccc23)c4cc(Br)c(O)c(Br)c4C | validation |
| 0 | CC(=O)NC1C(OC(=CC1N=C(N)N)C(=O)O)C(O)C(O)CO | validation |
| 0 | CNC(=O)CN(CCN(CCN(CC(=O)[O-])CC(=O)NC)CC(=O)[O-])CC(=O)[O-] | validation |
| 0 | C[N+]1(C)CCC(C1)OC(=O)C(O)(C2CCCC2)c3ccccc3 | validation |

| | | |
|---|---|---|
| 0 | OC(=O)CCCCCCCC(=O)O | validation |
| 0 | CN(C)C(=N)N=C(N)N | validation |
| 0 | CC(C)NCC(O)c1cc(O)cc(O)c1 | validation |
| 0 | C[C@@H](Cc1cc2CCN(CCCO)c2c(c1)C(=O)N)NCCOc3ccccc3OCC(F)(F)F | validation |
| 0 | Oc1cccc(c1)c2c3CCc(n3)c(c4cccc(O)c4)c5ccc([nH]5)c(c6cccc(O)c6)c7ccc(n7)c(c8C=Cc2[nH]8)c9cccc(O)c9 | validation |
| 0 | [O-]C(=O)CN(CCN(CC(=O)[O-])CC(=O)[O-])CCN(CC(=O)[O-])C(COCc1ccccc1)C(=O)[O-] | validation |
| 0 | NCCO | validation |
| 0 | OC[C@@H](O)[C@@H](CO)N1CCN(CC(=O)[O-])CCN(CC(=O)[O-])CCN(CC(=O)[O-])CC1 | validation |
| 0 | CCOC(=O)O[C@@]1(CCC2C3CCC4=CC(=O)C=C[C@]4(C)C3[C@@H](O)C[C@]12C)C(=O)OCCl | validation |
| 0 | NC(=NCc1cccc(I)c1)N | validation |
| 0 | CC12CCC3C(CCc4cc(O)ccc34)C1CCC2O | validation |
| 0 | CNc1ccc(cc1F)c2nc3ccc(O)cc3s2 | validation |
| 0 | OC(=O)c1cc(ccc1O)c2ccc(F)cc2F | validation |
| 0 | Clc1ccc([C@@H]2CSC(=C(C#N)n3ccnc3)S2)c(Cl)c1 | validation |
| 0 | ClC1C(Cl)C(Cl)C(Cl)C(Cl)C1Cl | validation |
| 0 | Cc1ccc(C)c(OCCCC(C)(C)C(=O)O)c1 | validation |
| 0 | Cc1cn[nH]c1 | validation |
| 0 | CN1CC(CC2C1Cc3c[nH]c4cccc2c34)C(=O)NC5(C)OC6(O)C7CCCN7C(=O)C(Cc8ccccc8)N6C5=O | validation |
| 0 | CN(C)C(=O)Cc1c(nc2ccc(C)cn12)c3ccc(C)cc3 | validation |
| 0 | CC(C)CCCC(C)CCCC(C)CCCC1(C)CCc2c(C)c(OC(=O)CCC(=O)OCCO)c(C)c(C)c2O1 | validation |
| 0 | CCN(CC)CC(=O)Nc1c(C)cccc1C | validation |
| 0 | CC(C)(C(=O)O)c1ccc(cc1)C(O)CCCN2CCC(CC2)C(O)(c3ccccc3)c4ccccc4 | validation |
| 0 | [O-][N+](=O)OCCNC(=O)c1cccnc1 | validation |
| 0 | CCOP(=O)(OCC)C(C)NC(=O)N(CCCl)N=O | validation |
| 0 | COc1nc(C)nc(Cl)c1NC2=NCCN2 | validation |
| 0 | OP(=O)([O-])[O-] | validation |
| 0 | CCOC(=O)C1=C(C)NC(=C(C1c2cccc(Cl)c2Cl)C(=O)OC)C | validation |
| 0 | CC(=O)NC(CS)C(=O)O | validation |
| 0 | COc1cc(CNC(=O)CCCCC=CC(C)C)ccc1O | validation |
| 0 | CC1CC2C3CCC4=CC(=O)C=CC4(C)C3(Cl)C(O)CC2(C)C1(OC(=O)c5occc5)C(=O)CCl | validation |
| 0 | Clc1ccc(C(Cn2ccnc2)OCc3csc4c(Cl)cccc34)c(Cl)c1 | validation |
| 0 | Nc1ncnc2c1c(nn2C3CCCN(C3)C(=O)C=C)c4ccc(Oc5ccccc5)cc4 | validation |
| 0 | CC(=O)C1CCC2C3CCC4=CC(=O)CCC4(C)C3CCC12C | validation |
| 0 | COCC(=O)Nc1c(I)c(C(=O)NCC(O)CO)c(I)c(C(=O)N(C)CC(O)CO)c1I | validation |
| 0 | CCC(=O)OC1(C(C)CC2C3CC(F)C4=CC(=O)C=CC4(C)C3(F)C(O)CC12C)C(=O)SCF | validation |
| 0 | Brc1ccc2NC(=O)CN=C(c3ccccn3)c2c1 | validation |
| 0 | CC(C(=O)O)c1cccc(Oc2ccccc2)c1 | validation |
| 0 | COc1ccc2C[C@H]3[C@@H]4CCC(=O)[C@@H]5Oc1c2[C@]45CCN3C | validation |
| 0 | [O-]C(=O)[O-] | validation |
| 0 | CC[C@@]12C=CCN3CC[C@@]4([C@H]13)[C@@H](N(C)c5cc(OC)c(cc45)[C@]6(C[C@@H]7C[C@@H](CN(C7)Cc8c6[nH]c9ccccc89)C(C)(F)F)C(=O)OC)[C@](O)([C@@H]2OC(=O)C)C(=O)OC | validation |
| 0 | COc1cccc2C(=O)c3c(O)c4CC(O)(CC(OC5CC(N)C(O)C(C)O5)c4c(O)c3C(=O)c12)C(=O)C | validation |
| 0 | Oc1ccc2CC3N(CC4CC4)CCC56C(Oc1c25)C(=O)CCC36O | validation |
| 0 | CCCCCCCCCCNCC=C | validation |
| 0 | CN1C(=O)C(O)N=C(c2ccccc2)c3cc(Cl)ccc13 | validation |
| 0 | CCOc1nc2cccc(C(=O)OC(C)OC(=O)OC3CCCCC3)c2n1Cc4ccc(cc4)c5ccccc5c6nn[nH]n6 | validation |
| 0 | CNC(=C[N+](=O)[O-])NCCSCc1csc(CN(C)C)n1 | validation |

| | | |
|---|---|---|
| 0 | CCNC(=O)N(CCCN(C)C)C(=O)C1CC2C(Cc3c[nH]c4cccc2c34)N(CC=C)C1 | validation |
| 0 | Clc1ccc2N(C3CCN(CCCN4C(=O)Nc5ccccc45)CC3)C(=O)Nc2c1 | validation |
| 0 | COc1ccc2c(OC3C[C@@H]4[C@@H](C3)C(=O)N(C)CCCC\C=C/[C@@H]5CC5(NC4=O)C(=O)NS(=O)(=O)C6CC6)cc(nc2c1C)c7nc(cs7)C(C)C | validation |
| 0 | NC(=O)N[C@@H](CCC(=O)O)C(=O)O | validation |
| 0 | CCCCC(F)(F)[C@@]1(O)CC[C@@H]2[C@@H](CCCCCCC(=O)O)C(=O)C[C@H]2O1 | validation |
| 0 | Oc1ccc2c(Oc3cc(O)ccc3C24OC(=O)c5ccccc45)c1 | validation |
| 0 | C[C@]12CC[C@H]3[C@@H](CC=C4C[C@@H](O)CC[C@]34C)[C@@H]1CC=C2c5cccnc5 | validation |
| 0 | [O-][N+](=O)OCC(CO[N+](=O)[O-])O[N+](=O)[O-] | validation |
| 0 | NCCCC(O)(P(=O)(O)O)P(=O)(O)O | validation |
| 0 | COCCNC(=O)CN(CCN(CCN(CC(=O)[O-])CC(=O)NCCOC)CC(=O)[O-])CC(=O)[O-] | validation |
| 0 | CCC(=O)OC1([C@H](C)C[C@H]2[C@@H]3C[C@H](F)C4=CC(=O)C=C[C@]4(C)C3(F)[C@@H](O)C[C@]12C)C(=O)SCF | validation |
| 0 | C(N1CCCNCCNCCCNCC1)c2ccc(CN3CCCNCCNCCCNCC3)cc2 | validation |
| 0 | NC(=N)c1ccc(OCCCCCOc2ccc(cc2)C(=N)N)cc1 | validation |
| 0 | CN1C(=O)N(CCCCC(=O)C)C(=O)c2c1ncn2C | validation |
| 0 | Fc1ccccc1C2=NCC(=S)N(CC(F)(F)F)c3ccc(Cl)cc23 | validation |
| 0 | CCC(CO)NC(=O)C1CN(C)C2Cc3c[nH]c4cccc(C2=C1)c34 | validation |
| 0 | CC1CC2C3CCC4=CC(=O)C=CC4(C)C3(F)C(O)CC2(C)C1C(=O)CO | validation |
| 0 | OC(=O)C(=O)Nc1cc(cc(NC(=O)C(=O)O)c1Cl)C#N | validation |
| 0 | CCCCCN(C)CCC(O)(P(=O)(O)O)P(=O)(O)O | validation |
| 0 | [O-]C(=O)CN(CC(=O)[O-])c1sc(C(=O)[O-])c(CC(=O)[O-])c1C#N | validation |
| 0 | [O-]C(=O)CN(CCN(CC(=O)[O-])CC(=O)[O-])CCN(CC(=O)[O-])CC(=O)[O-] | validation |
| 0 | CC1OC(CC(N)C1O)OC2CC(O)(Cc3c(O)c4C(=O)c5ccccc5C(=O)c4c(O)c23)C(=O)C | validation |
| 0 | CC(O)(P(=O)(O)O)P(=O)(O)O | validation |
| 0 | CC(C)(C)NC(=O)C1CCC2C3CCC4NC(=O)C=CC4(C)C3CCC12C | validation |
| 0 | CCOc1cc(CC(=O)NC(CC(C)C)c2ccccc2N3CCCCC3)ccc1C(=O)O | validation |
| 0 | CC(C[N+](C)(C)C)OC(=O)N | validation |
| 0 | C[C@@H]1CCN(CCN1C(=O)c2cc(C)ccc2n3nccn3)c4oc5ccc(Cl)cc5n4 | validation |
| 0 | O[C@H]1O[C@H](COS(=O)(=O)O)[C@@H](O[C@@H]2O[C@H]([C@@H](O)[C@H](O)[C@H]2OS(=O)(=O)O)C(=O)O)[C@H](O)[C@H]1NS(=O)(=O)O | validation |
| 0 | Cc1ccc(cc1Cc2ccc(s2)c3ccc(F)cc3)[C@@H]4O[C@H](CO)[C@@H](O)[C@H](O)[C@H]4O | validation |
| 0 | [O-][N+](=O)c1ccc2NC(=O)CN=C(c3ccccc3Cl)c2c1 | validation |
| 0 | CC(C)Cc1ccc(cc1)C(C)C(=O)O | validation |
| 0 | CCCSc1ccc2nc(NC(=O)OC)[nH]c2c1 | validation |
| 0 | CCN(CC)c1ccc(cc1)C(=C2C=CC(=[N+](CC)CC)C=C2)c3cc(ccc3S(=O)(=O)O)S(=O)(=O)O | validation |
| 0 | N#Cc1ccc(cc1)C(c2ccc(cc2)C#N)n3cncn3 | validation |
| 0 | CC(C)COCC(CN(Cc1ccccc1)c2ccccc2)N3CCCC3 | validation |
| 0 | Cc1ncc[nH]1 | validation |
| 0 | CC(CCCC(C)(C)O)C1CCC2C(=CC=C3CC(O)CC(O)C3=C)CCCC12C | validation |
| 0 | CCCCN1CCCCC1C(=O)Nc2c(C)cccc2C | validation |
| 0 | CN1CC(=O)N2[C@H](Cc3c([nH]c4ccccc34)[C@H]2c5ccc6OCOc6c5)C1=O | validation |
| 0 | COC(=O)C=CC(=O)O | validation |
| 0 | CC(C)(C)NCC(O)c1cc(O)cc(O)c1 | validation |
| 0 | C[C@]12CC[C@H]3[C@@H](CC(=C)C4=CC(=O)C=C[C@]34C)[C@@H]1CCC2=O | validation |
| 0 | CN1C[C@@H]2C(C1)c3ccccc3Oc4ccc(Cl)cc24 | validation |
| 0 | CC(C)N(CC[C@H](c1ccccc1)c2cc(CO)ccc2OC(=O)C(C)C)C(C)C | validation |
| 0 | COc1ccc2c(c1)c(CC(=O)O)c(C)n2C(=O)c3ccc(Cl)cc3 | validation |
| 0 | CC(C)N(CCC(c1ccccc1)c2cc(C)ccc2O)C(C)C | validation |
| 0 | CNC(=O)c1ccccc1Sc2ccc3c(C=Cc4ccccn4)n[nH]c3c2 | validation |

| | | |
|---|---|---|
| 0 | O[C@H]1CO[C@@H]2[C@@H](CO[C@H]12)O[N+](=O)[O-] | validation |
| 0 | CCC12CCC3C(CCC4=CC(=O)CCC34)C1CCC2(O)C#C | validation |
| 0 | C[C@H](O)C1C2[C@@H](C)C(=C(N2C1=O)C(=O)O)S[C@@H]3CN[C@@H](C3)C(=O)N(C)C | validation |
| 0 | CC(C)[N+]1(C)C2CCC1CC(C2)OC(=O)C(CO)c3ccccc3 | validation |
| 0 | CCC(=O)NC[C@@H]1C[C@@H]1c2cccc3OCCc23 | validation |
| 0 | CCC(=O)NCC[C@@H]1CCc2ccc3OCCc3c12 | validation |
| 0 | OCCCC(=O)O | validation |
| 0 | FC(F)OC(F)C(F)(F)F | validation |
| 0 | CC1CC2C3CCC4=CC(=O)C=CC4(C)[C@@]3(F)C(O)CC2(C)[C@@]1(O)C(=O)CCl | validation |
| 0 | S=P(N1CC1)(N2CC2)N3CC3 | validation |
| 0 | CC1C(NC(=O)C(=NOC(C)(C)C(=O)O)c2csc(N)n2)C(=O)N1S(=O)(=O)O | validation |
| 0 | CC1CNCCc2ccc(Cl)cc12 | validation |
| 0 | OC1CCC2(O)C3Cc4ccc(O)c5OC1C2(CCN3CC6CCC6)c45 | validation |
| 0 | NC(=NC(=O)Cc1c(Cl)cccc1Cl)N | validation |
| 0 | CCOCCn1c(nc2ccccc12)N3CCCN(C)CC3 | validation |
| 0 | COc1ccc2[n-]c(nc2c1)S(=O)Cc3ncc(C)c(OC)c3C | validation |
| 0 | CC(N(O)C(=O)N)c1cc2ccccc2s1 | validation |
| 0 | CCCCCCCN(CC)CCCC(O)c1ccc(NS(=O)(=O)C)cc1 | validation |
| 0 | CC1(C)N=C(N)N=C(N)N1OCCCOc2cc(Cl)c(Cl)cc2Cl | validation |
| 0 | CNC(=O)c1ccc(cc1F)N2C(=S)N(C(=O)C2(C)C)c3ccc(C#N)c(c3)C(F)(F)F | validation |
| 0 | COC12CCC3(CC1C(C)(O)C(C)(C)C)C4Cc5ccc(O)c6OC2C3(CCN4CC7CC7)c56 | validation |
| 0 | CN1C(=O)C=C(N2CCCC(N)C2)N(Cc3ccccc3C#N)C1=O | validation |
| 0 | Cc1onc(c1C(=O)NC2C3SC(C)(C)C(N3C2=O)C(=O)[O-])c4c(Cl)cccc4Cl | validation |
| 0 | CNNCc1ccc(cc1)C(=O)NC(C)C | validation |
| 0 | CCCCC(=O)O[C@@]1(CCC2C3CCC4=CC(=O)CCC4(C)C3C(O)CC12C)C(=O)CO | validation |
| 0 | OC(c1ccccc1)(c2ccccc2)C34CC[N+](CCOCc5ccccc5)(CC3)CC4 | validation |
| 0 | CN1C2CC(CC1C3OC23)OC(=O)C(CO)c4ccccc4 | validation |
| 0 | CC(C)(C)NCC(O)c1ccc(O)c(CO)c1 | validation |
| 0 | CCOC(=O)N1CCC(=C2c3ccc(Cl)cc3CCc4cccnc24)CC1 | validation |
| 0 | NC(=Nc1nc(CSCCC(=NS(=O)(=O)N)N)cs1)N | validation |
| 0 | NS(=O)(=O)c1cc2c(NCNS2(=O)=O)cc1Cl | validation |
| 0 | CC(C)N1CCN(CC1)c2ccc(OCC3COC(Cn4cncn4)(O3)c5ccc(Cl)cc5Cl)cc2 | validation |
| 0 | CCCC(C)(COC(=O)N)COC(=O)NC(C)C | validation |
| 0 | ClCC1CO1 | validation |
| 0 | FC(F)(F)C(F)(F)C(F)(F)F | validation |
| 0 | CC12CCC(=O)C=C1CCC3C4CCC(O)(C(=O)CO)C4(C)CC(O)C23 | validation |
| 0 | CN(C)CCc1c[nH]c2ccc(Cn3cncn3)cc12 | validation |
| 0 | CC(C(=O)O)c1ccc(s1)C(=O)c2ccccc2 | validation |
| 0 | COc1ccc(CNc2nc(ncc2C(=O)NCc3ncccn3)N4CCC[C@H]4CO)cc1Cl | validation |
| 0 | CC(O)CN1CCN(CC(=O)[O-])CCN(CC(=O)[O-])CCN(CC(=O)[O-])CC1 | validation |
| 0 | CC(C)C(C)C=CC(C)C1CCC2C(=CC=C3CC(O)CC(O)C3=C)CCCC12C | validation |
| 0 | CCCCCC(O)C=CC1C(O)CC(O)C1CC=CCCCC(=O)O | validation |
| 0 | C[C@@H]1C[C@H]2[C@@H]3C[C@H](F)C4=CC(=O)C=C[C@]4(C)[C@@]3(F)[C@@H](O)C[C@]2(C)[C@@]1(OC(=O)c5occc5)C(=O)SCF | validation |
| 0 | Clc1ccc(COC(Cn2ccnc2)c3ccc(Cl)cc3Cl)c(Cl)c1 | validation |
| 0 | CN(C)[C@H]1[C@@H]2CC3C(=C(O)c4c(O)cccc4[C@@]3(C)O)C(=O)[C@]2(O)C(=C(C(=O)NCNCCCC[C@H](N)C(=O)O)C1=O)O | validation |
| 0 | CC(C)(C)NCC(O)c1ccc(O)c(CO)n1 | validation |
| 0 | OP(=O)(O)C(Cl)(Cl)P(=O)(O)O | validation |
| 0 | OS(=O)(=O)CCS | validation |
| 0 | COc1cc2nc(nc(N)c2cc1OC)N3CCN(CC3)C(=O)c4occc4 | validation |
| 0 | CCCC1CC(N(C)C1)C(=O)NC(C(C)Cl)C2OC(SC)C(OP(=O)(O)O)C(O)C2O | validation |
| 0 | O=C(CCCc1ccccc1)OCC(COC(=O)CCCc2ccccc2)OC(=O)CCCc3ccccc3 | validation |
| 0 | CC1CCN(CC1N(C)c2ncnc3[nH]ccc23)C(=O)CC#N | validation |

| | | |
|---|---|---|
| 0 | CCOC(=O)c1ccc(nc1)C#Cc2ccc3SCCC(C)(C)c3c2 | validation |
| 0 | C[N+]1(C)C2CC(CC1C3OC23)OC(=O)C(O)(c4cccs4)c5cccs5 | validation |
| 0 | Clc1ccc2C(=C3CCNCC3)c4ncccc4CCc2c1 | validation |
| 0 | Nc1nc(N)c2nc(CC(CC#C)c3ccc(cc3)C(=O)N[C@@H](CCC(=O)O)C(=O)O)cnc2n1 | validation |
| 0 | CCC1C(Cc2cncn2C)COC1=O | validation |
| 0 | CN(CCCl)CCCl | validation |
| 0 | CC12Cc3cnoc3C=C1CCC4C2CCC5(C)C4CCC5(O)C#C | validation |
| 0 | CC1CCN(C(C1)C(=O)O)C(=O)C(CCCN=C(N)N)NS(=O)(=O)c2cccc3CC(C)CNc23 | validation |
| 0 | NC(=O)C(C1CCN(CCc2ccc3OCCc3c2)C1)(c4ccccc4)c5ccccc5 | validation |
| 0 | Cc1ccc(cc1)c2cc(nn2c3ccc(cc3)S(=O)(=O)N)C(F)(F)F | validation |
| 0 | CC#CCn1c(nc2N(C)C(=O)N(Cc3nc(C)c4ccccc4n3)C(=O)c12)N5CCC[C@@H](N)C5 | validation |
| 0 | CC(C(=O)O)c1cccc(c1)C(=O)c2ccccc2 | validation |
| 0 | O=C(OC1CC2CC3CC(C1)N2CC3=O)c4c[nH]c5ccccc45 | validation |
| 0 | NCC(CC(=O)O)c1ccc(Cl)cc1 | validation |
| 0 | CCCCCCOC(=O)CCC(=O)CN | validation |
| 0 | Cc1nnc2CN=C(c3ccccc3)c4cc(Cl)ccc4n12 | validation |
| 0 | Oc1ccc2CC3N(CC4CC4)CCC56C(Oc1c25)C(=C)CCC36O | validation |
| 0 | Clc1ccc(s1)C(=O)NCC2CN(C(=O)O2)c3ccc(cc3)N4CCOCC4=O | validation |
| 0 | CC(O)C(=O)Nc1c(I)c(C(=O)NC(CO)CO)c(I)c(C(=O)NC(CO)CO)c1I | validation |
| 0 | COc1ccccc1OCC(O)CN2CCN(CC(=O)Nc3c(C)cccc3C)CC2 | validation |
| 0 | CCC(C)[C@H]1O[C@]2(CC[C@@H]1C)CC3C[C@@H](C\C=C(/C)\[C@@H](O[C@H]4C[C@H](OC)[C@@H](OC5C[C@H](OC)[C@@H](O)[C@H](C)O5)[C@H](C)O4)[C@@H](C)\C=C\C=C\6/CO[C@@H]7[C@H](O)C(=C[C@@H](C(=O)O3)[C@]67O)C)O2 | validation |
| 0 | COc1ccc(CC(C)(C)NCC(O)c2cc(O)cc3NC(=O)COc23)cc1 | validation |
| 0 | Brc1c(NC2=NCCN2)ccc3nccnc13 | validation |
| 0 | C\C=C(\C)/C(=O)O[C@H]1C(=C[C@@]23[C@H](C)C[C@@H]4[C@H]([C@H](C=C(CO)[C@@H](O)[C@]12O)C3=O)C4(C)C)C | validation |
| 0 | CC(C)OC(=O)CCCC=CCC1C(O)CC(O)C1CCC(O)CCc2ccccc2 | validation |
| 0 | CC(=O)OC1CC2CCC3C(CCC4(C)C3CC(C4OC(=O)C)[N+]5(C)CCCCC5)C2(C)CC1[N+]6(C)CCCCC6 | validation |
| 0 | O=C(OOC(=O)c1ccccc1)c2ccccc2 | validation |
| 0 | CN1CCCC1c2cccnc2 | validation |
| 0 | CC1CC2C3CCC(O)(C(=O)C)C3(C)CC(O)C2(F)C4(C)C=CC(=O)C=C14 | validation |
| 0 | CC(C)(C(=O)c1cccnc1)c2cccnc2 | validation |
| 0 | Cc1[nH]cnc1CN2CCc3c(C2=O)c4ccccc4n3C | validation |
| 0 | CN(C)CCCOc1nn(Cc2ccccc2)c3ccccc13 | validation |
| 0 | COC1C(O)C(O)C(OCC2OC(OC3C(O)C(O)C(C)OC3CO)C(O)C(O)C2OC4OC(CO)C(OC)C(O)C4OCCO)OC1CO | validation |
| 0 | CCCCCCCC(=O)CCC1C(O)CC(O)C1CC=CCCCC(=O)O | validation |
| 0 | CN1CCC23C4Oc5c(O)ccc(CC1C2C=CC4O)c35 | validation |
| 0 | CC1CN(CCC1(C(=O)O)c2ccccc2)C3CCC(CC3)(C#N)c4ccc(F)cc4 | validation |
| 0 | COc1c2OC(=O)C=Cc2cc3ccoc13 | validation |
| 0 | OCCN(C(=O)CO)c1c(I)c(C(=O)NCC(O)CO)c(I)c(C(=O)NCC(O)CO)c1I | validation |
| 0 | OC12CC3CC(C1)CC(C3)(C2)NCC(=O)N4CCCC4C#N | validation |
| 0 | CCO\N=C(/C(=O)NC1C2SCC(=C(N2C1=O)C(=O)O)Sc3nc(cs3)c4cc[n+](C)cc4)\c5nsc(NP(=O)(O)[O-])n5 | validation |
| 0 | C[C@@H]1CC[C@@]23CCC(=O)[C@H]2[C@]1(C)[C@@H](C[C@@](C)(C=C)[C@@H](O)[C@@H]3C)OC(=O)CSC4C[C@H]5CC[C@@H](C4)N5C | validation |
| 0 | O=C1N(C=C(C=C1c2ccccc2C#N)c3ccccn3)c4ccccc4 | validation |
| 0 | FC(F)OC(Cl)C(F)(F)F | validation |
| 0 | CC(=O)N(CC(O)CO)c1c(I)c(C(=O)NCC(O)CO)c(I)c(C(=O)NCC(O)CO)c1I | validation |
| 0 | CC[C@@H]1C=C(C)C[C@H](C)C[C@H](OC)[C@H]2O[C@](O)([C@H](C)C[C@@H]2OC)C(=O)C(=O)N3CCCC[C@H]3C(=O)O[C@@H]([C@H](C)[C@@H](O)CC1=O)C(=C[C@@H]4CC[C@H](Cl)[C@@H](C4)OC)C | validation |

| | | |
|---|---|---|
| 0 | COc1c(N2CC3CCCNC3C2)c(F)cc4C(=O)C(=CN(C5CC5)c14)C(=O)O | validation |
| 0 | Cc1nnc2CN=C(c3ccccc3Cl)c4cc(Cl)ccc4n12 | validation |
| 0 | Cc1onc(c1)C(=O)NNCc2ccccc2 | validation |
| 0 | Cc1c(c2ccc(O)cc2)n(Cc3ccc(OCCN4CCCCCC4)cc3)c5ccc(O)cc15 | validation |
| 0 | CC#CC1(O)CCC2C3CCC4=CC(=O)CCC4=C3C(CC12C)c5ccc(cc5)N(C)C | validation |
| 0 | CC(C)c1nc(CN(C)C(=O)NC(CCN2CCOCC2)C(=O)NC(CCC(Cc3ccccc3)NC(=O)OCc4cncs4)Cc5ccccc5)cs1 | validation |
| 0 | CNCC[C@@H](Oc1ccccc1C)c2ccccc2 | validation |
| 0 | NC(CCC(=O)O)C=C | validation |
| 0 | NC12CC3CC(CC(C3)C1)C2 | validation |
| 0 | CCCC(=O)OC1(C(C)CC2C3CCC4=CC(=O)C=CC4(C)C3(F)C(=O)CC12C)C(=O)CCl | validation |
| 0 | CC(C)(C)NCC(O)COc1ccccc1C2CCCC2 | validation |
| 0 | CCCN(CCc1cccs1)C2CCc3c(O)cccc3C2 | validation |
| 0 | CN(C)CC(c1ccc(O)cc1)C2(O)CCCCC2 | validation |
| 0 | COC1C(CC2CN3CCc4c([nH]c5cc(OC)ccc45)C3CC2C1C(=O)OC)OC(=O)C=Cc6cc(OC)c(OC)c(OC)c6 | validation |
| 0 | Cc1cc(C)c(C=C2C(=O)Nc3ccccc23)[nH]1 | validation |
| 0 | O=C(O[C@H]1CN2CCC1CC2)N3CCc4ccccc4[C@@H]3c5ccccc5 | validation |
| 0 | CCCCN(CCCC)CCC(O)c1cc2c(Cl)cc(Cl)cc2c3cc(ccc13)C(F)(F)F | validation |
| 0 | Cc1ncc(COP(=O)([O-])[O-])c(CN(CCN(CC(=O)[O-])Cc2c([O-])c(C)ncc2COP(=O)([O-])[O-])CC(=O)[O-])c1[O-] | validation |
| 0 | CCC(=O)O[C@@]1([C@@H](C)C[C@H]2[C@@H]3CCC4=CC(=O)C=C[C@]4(C)[C@@]3(Cl)[C@@H](O)C[C@]12C)C(=O)CO | validation |
| 0 | CCOc1ccccc1O[C@H]([C@@H]2CNCCO2)c3ccccc3 | validation |
| 0 | CCc1ccc(CCOc2ccc(CC3SC(=O)NC3=O)cc2)nc1 | validation |
| 0 | Cc1ccc(cc1)N(CC2=NCCN2)c3cccc(O)c3 | validation |
| 0 | COCCOC(=O)NCCCC[C@H](NC(=O)OCCOC)C(=O)NCCCCCCCOP(=O)(C)O | validation |
| 0 | Fc1ccc(cc1)C(CCCN2CCC(CC2)N3C(=O)Nc4ccccc34)c5ccc(F)cc5 | validation |
| 0 | CCCCCC(O)C=CC1C(O)CC2OC(=CCCCC(=O)O)CC12 | validation |
| 0 | CCN(CC)CCOC(=O)C1(CCCCC1)C2CCCCC2 | validation |
| 0 | CC(C)(C)c1cc(c(NC(=O)C2=CNc3ccccc3C2=O)cc1O)C(C)(C)C | validation |
| 0 | CCC(=O)N(C1CCN(CCc2ccccc2)CC1)c3ccccc3 | validation |
| 0 | CCC(=O)N(c1ccccc1)C2(COC)CCN(CCc3cccs3)CC2 | validation |
| 0 | C[C@@]1(COc2ccc(cc2)N3CCC(CC3)Oc4ccc(OC(F)(F)F)cc4)Cn5cc(nc5O1)[N+](=O)[O-] | validation |
| 0 | CC(C)(C#N)c1cc(Cn2cncn2)cc(c1)C(C)(C)C#N | validation |
| 0 | COC(=O)C1=C(C)NC(=C(C1c2cccc3nonc23)C(=O)OC(C)C)C | validation |
| 0 | CCCC1OC2CC3C4CCC5=CC(=O)C=CC5(C)C4C(O)CC3(C)C2(O1)C(=O)C | validation |
| 0 | Cc1nccn1CC2CCc3c(C2=O)c4ccccc4n3C | validation |
| 0 | COc1cc2c(CCNC23CSC4C5C6N(C)C(Cc7cc(C)c(OC)c(O)c67)C(O)N5C(COC3=O)c8c9OCOc9c(C)c(OC(=O)C)c48)cc1O | validation |
| 0 | CC(O)C(C)C1OC1CC2COC(CC(=CC(=O)OCCCCCCCCC(=O)O)C)C(O)C2O | validation |
| 0 | CN(C)CCC=C1c2ccccc2COc3ccc(CC(=O)O)cc13 | validation |
| 0 | CCCCCCCC(=O)NC(CN1CCCC1)C(O)c2ccc3OCCOc3c2 | validation |
| 0 | CC1=C(C=C(C#N)C(=O)N1)c2ccncc2 | validation |
| 0 | CC(Cc1ccccc1)N(C)CC#C | validation |
| 0 | CCC[C@H](NC(=O)[C@@H]1[C@H]2CCC[C@H]2CN1C(=O)[C@@H](NC(=O)[C@@H](NC(=O)c3cnccn3)C4CCCCC4)C(C)(C)C)C(=O)C(=O)NC5CC5 | validation |
| 0 | COc1ccc2CC3C4CCCCC4(CCN3C)c2c1 | validation |
| 0 | COC(=O)C1=C(C)NC(=C(C1c2ccccc2[N+](=O)[O-])C(=O)OCC(C)C)C | validation |
| 0 | CCN1CCN(C(=O)NC(C(=O)NC2C3SC(C)(C)C(N3C2=O)C(=O)[O-])c4ccccc4)C(=O)C1=O | validation |
| 0 | COc1cc(Br)c(C[N+]2(CCOCCC3CCC4CC3C4(C)C)CCOCC2)cc1OC | validation |
| 0 | CC1CC2C3CCC4=CC(=O)C=CC4(C)C3(F)C(O)CC2(C)C1(O)C(=O)CO | validation |

| | | |
|---|---|---|
| 0 | CO[C@@H]1O[C@@H](COS(=O)(=O)O)[C@H](O[C@@H]2O[C@@H]([C@H](OC)[C@@H](O)[C@@H]2OS(=O)(=O)O)C(=O)[O-])[C@@H]([O-])[C@@H]1NOS(=O)(=O)O | validation |
| 0 | CCOc1ccccc1OCCN[C@@H](C)Cc2ccc(OC)c(c2)S(=O)(=O)N | validation |
| 0 | CC1OC(CC(O)C1O)OC2C(O)CC(OC3C(O)CC(OC4CCC5(C)C(CCC6C5CC(O)C7(C)C(CCC67O)C8=CC(=O)OC8)C4)OC3C)OC2C | validation |

**Table S2 The optimized parameters for SVM and kNN models**

| Fingerprint | Parameters for SVM | | Parameter for kNN |
|---|---|---|---|
| | C | g | k |
| Estate | 13 | 0.55 | 19 |
| ExtendFP | 19 | 0.003 | 21 |
| FP | 17 | 0.0005 | 7 |
| MACCS | 10 | 0.55 | 3 |
| KRFP | 9 | 0.005 | 9 |
| Pubchem | 5 | 0.009 | 9 |
| SubFP | 3 | 0.3 | 11 |
